# scEpiAge: an age predictor highlighting single-cell ageing heterogeneity in mouse blood

**DOI:** 10.1038/s41467-024-51833-5

**Published:** 2024-08-31

**Authors:** Marc Jan Bonder, Stephen J. Clark, Felix Krueger, Siyuan Luo, João Agostinho de Sousa, Aida M. Hashtroud, Thomas M. Stubbs, Anne-Katrien Stark, Steffen Rulands, Oliver Stegle, Wolf Reik, Ferdinand von Meyenn

**Affiliations:** 1 Division of Computational Genomics and Systems Genetics, German Cancer Research Center, Heidelberg, Germany; 2 Genome Biology Unit, European Molecular Biology Laboratory, Heidelberg, Germany; 3 Department of Genetics, University of Groningen, University Medical Center Groningen, Groningen, the Netherlands; 4 Oncode Institute, Utrecht, The Netherlands; 5 Altos Labs, Cambridge Institute of Science, Cambridge, UK; 6 Epigenetics Programme, The Babraham Institute, Cambridge, UK; 7 Bioinformatics Group, The Babraham Institute, Cambridge, UK; 8 Laboratory of Nutrition and Metabolic Epigenetics, Department of Health Sciences and Technology, ETH Zurich, Zurich, Switzerland; 9 Max Planck Institute for the Physics of Complex Systems, Dresden, Germany; 10 Arnold Sommerfeld Center for Theoretical Physics and Center for NanoScience, Department of Physics, Ludwig-Maximilians-Universität, Munich, Germany; 11 Chronomics Limited, London, UK; 12 Immunology Programme, The Babraham Institute, Cambridge, UK; 13 Wellcome Trust Sanger Institute, Hinxton, Cambridge, UK; 14 Department of Medical and Molecular Genetics, King’s College London, London, UK

**Keywords:** Ageing, Epigenetics, Biomarkers

## Abstract

Ageing is the accumulation of changes and decline of function of organisms over time. The concept and biomarkers of biological age have been established, notably DNA methylation-based clocks. The emergence of single-cell DNA methylation profiling methods opens the possibility of studying the biological age of individual cells. Here, we generate a large single-cell DNA methylation and transcriptome dataset from mouse peripheral blood samples, spanning a broad range of ages. The number of genes expressed increases with age, but gene-specific changes are small. We next develop scEpiAge, a single-cell DNA methylation age predictor, which can accurately predict age in (very sparse) publicly available datasets, and also in single cells. DNA methylation age distribution is wider than technically expected, indicating epigenetic age heterogeneity and functional differences. Our work provides a foundation for single-cell and sparse data epigenetic age predictors, validates their functionality and highlights epigenetic heterogeneity during ageing.

## Abstract (web summary)

Here, the authors generate a single-cell DNA methylation and transcriptome dataset from mouse peripheral blood samples, spanning a broad range of ages. They next develop scEpiAge, a single-cell DNA methylation age predictor.

## Introduction

The observation that specific DNA methylation patterns at CpG sites (hereafter DNAme) correlate robustly with age has led to the development of statistical models trained to predict age using DNAme measurements at a few hundred genomic loci^1–3^. These models, termed epigenetic or DNAme clocks, are capable of great accuracy, demonstrating their importance in the ageing field^2^. DNAme clocks have been created for a variety of species, including human, mouse^4–6^, and more recently across multiple species, demonstrating the evolutionary conservation of the DNAme ageing process^7^. Noteworthy, while these models are trained based on chronological age, they do predict biological age, i.e., the biological functional decline associated with ageing, which can be dissociated from chronological age and is linked to disease risk and progression^8–10^. These results illustrate the medical utility of DNAme ageing clocks and their relevance for measuring the effects of perturbations, such as drugs and other interventions. However, many DNAme–age associations are potentially confounded by changes in cell composition, particularly in blood where proportions of white blood cells are known to vary with age^11^. Additionally, within a given tissue or cell type, it is not known whether DNAme ageing is a cell-intrinsic process, such that all cells age at the same rate, or whether the ageing process is inherently heterogeneous and, as such, a cell population phenomenon. This underscores the need for better understanding and the relevance to develop single-cell age predictors and analysis. Additionally, the potential to use these age predictors in genetic or drug perturbation assays can facilitate massively parallel single-cell screens with biological age as a readout.

The possibility to profile DNAme in single cells genome-wide has become available in the last few years^12–15^, but the datasets generated by these methods differ substantially from those generated by bulk approaches in three key aspects. First, single-cell DNAme data is almost entirely binary—i.e., methylation values are either 0 or 100% for any given cytosine. Second, the datasets are very sparse with typically >90% missing values. Finally, the genomic coverage is essentially random, such that a dataset of a few hundred cells will contain no or only very few genomic loci with information from every single cell. These properties represent major challenges for generating age predictors using current approaches, which have relied on datasets with deep coverage at a consistent set of CpG sites. In earlier work, where muscle stem cells from young and old mice were studied^16^, we addressed this issue by aggregating the single-cell datasets and then retraining the original model^5^, only including sites covered in the single-cell dataset. While this approach was feasible, it is not scalable and only applicable if several preconditions are fulfilled.

Here, we develop an approach termed scEpiAge which addresses these challenges by taking a different approach to traditional epigenetic clocks. Our method works by reversing the standard elastic net regression setup, predicting DNAme from age as opposed to age given DNAme and crucially does not require the same set of CpG sites to be covered in each cell, whilst explicitly leveraging redundancy and modelling different possible relationships between age and DNAme. A recent study^17^ built a similar model, but the authors could only validate their predictions on a small dataset of publicly available adult mouse hepatocytes. In this study, we therefore also generate a large single-cell dataset from peripheral blood cells of mice spanning a broad range of ages (10–101 weeks), using scM&T-seq^18^ to profile single-cell methylomes and transcriptomes from the same cells.

We show that scEpiAge allows us to predict epigenetic age more accurately in single cells than previous methods and can also be applied to bulk methylation data, including low-depth bisulfite sequencing. Further, our analyses reveal cell type-dependent biological variation in the epigenetic age of mouse blood cells of a given chronological age. Overall, we present a robust single-cell age estimator in the mouse and develop a methodological foundation for better epigenetic age predictors.

## Results

### Ageing-related changes in expression and DNA methylation in blood-derived single cells

**Fig. 1:**
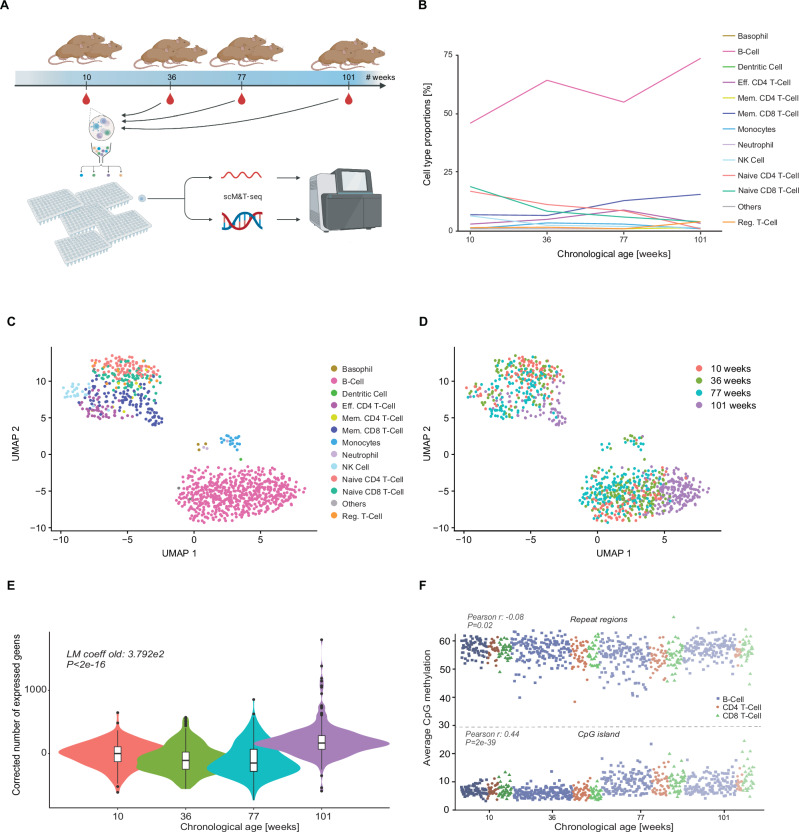
Study overview and data exploration. **A** Illustration of the data collected for this study. We collected blood at 4 time points (10, 36, 77 and 101 weeks of age) from three mice each, FACS sorted single cells into 96-well plates and performed scM&T-seq^18^ on 1055 cells in total. Created with BioRender.com released under a Creative Commons Attribution-NonCommercial-NoDerivs 4.0 International license. **B** Cell type composition overview stratified by chronological age. Lines depict the average percentages. **C**, **D** Exploratory UMAP figures of the single-cell expression data: **C** Cell type annotated UMAP, and **D** UMAP annotated by chronological age. **E** Violin plots and boxplots showing the number of genes expressed/detected in each cell at the different mouse ages. Boxplots show median levels and the first and third quartile, whiskers show 1.5× the interquartile range. Statistics shown are from a linear model testing the number of expressed genes in the 101-week-old mice versus the younger ages. **F** Average single-cell DNAme levels in repeat regions (top half) and CGIs (bottom half). Shape and colour represent the cell type (blue squares B-cell, orange dots CD4+ T-cell, green triangles CD8+ T-cell), ordered by age. Statistics shown are from Pearson correlations assessing the link between DNAme in repeat regions versus age (top), and DNAme in CGIs and age (bottom).

We collected peripheral blood from mice spanning ages from 10 to 101 weeks (Fig. 1A, Supplementary Data 1). Following red blood cell lysis, we sorted individual cells into 96-well plates containing scM&T-seq lysis buffer. Subsequently, we performed scM&T-seq^18^ and generated paired single-cell methylomes and transcriptomes from 1055 cells, of which 853 passed methylation quality control (QC) filters, 981 passed expression QC filters and 823 passed filters on both (Supplementary Data 1).

As a first step, we used the single-cell transcriptome data to perform combined de novo and reference-based cell type annotation (see “Methods” for details). This resulted in a cell type classification and identification of the major categories of blood cell types. The cell type proportions reflect the expected distribution of B-, T-, and other blood cells (Fig. 1B)^19^. Examining the cell type proportions as a function of age, using propeller^20^, did not show any significant ageing effect. Suggestively, we observe an increase in the B-cells proportion with age, agreeing with prior reports^19^, but this change was not significant in our sample set, probably due to the lower sample and cell numbers.

Dimensionality reduction of the single-cell transcriptome data showed clear segregation by cell type and not by age or animal (Fig. 1C, D). The DNAme data did not show clear separations by age, cell type, or animal (Supplementary Fig. 1). This is likely explained by the sparse CpG coverage of the single-cell data. While we obtained data for approximately 1 million CpG sites in each cell, the number of CpGs covered between cells did drop markedly when jointly analysing multiple cells, even when we analysed the data at genome feature level (for instance, at promoters or enhancers). The global analysis of gene expression highlights that there is a significant difference between the number of genes expressed in samples from younger ages (10, 36, and 77 weeks) vs from older mice (101 weeks; Likelihood-ratio test (LRT) *p* < 2e-16; Fig. 1E), driving the separation observed in the UMAP plots (Fig. 1C, D). This effect is consistent in the three major cell types (Supplementary Fig. 2A–C), and we were able to replicate the effect in the four tissues of the Tabula Muris Senis study^21^ (Supplementary Data 2), but we also observed significant opposite effects depending on the tissue. Given this incomplete replication, we also assessed the effect in a large human PBMC cohort, the OneK1K dataset^22^, where we also found that samples below 55 years of age (roughly equivalent to under 77 weeks in mouse) have significantly fewer genes expressed (LRT *p* < 2e-16; Supplementary Fig. 2D) as compared to samples over 64 (roughly equivalent to over 101 weeks of age in mice).

When comparing cells at the level of DNAme at repeat regions or CpG islands (CGIs; Fig. 1F), we observe limited variation between the cells over cell types and age, indicating high-quality data (see “Methods” for details). Interestingly, at shared CGIs, DNAme levels increased with age (*r* = 0.44, *p* < 2e-39), a phenomenon that could be replicated using data from sorted immune cells (*r* = 0.86, *p* < 9e-10; see “Methods” for details; Supplementary Fig. 3A). In contrast, in repeat regions we observed a weak but significant negative correlation with age in our single-cell data (Pearson *r* = −0.08, *p* = 0.02, see “Methods” for details), and this effect was even stronger in the bulk datasets (Pearson *r* = −0.45, *p* = 0.01; see “Methods” for details; Supplementary Fig. 3B).

We next assessed transcriptional differences at individual genes, using MAST (see “Methods” for details), and DNAme differences at the level of individual enhancers and promoters using a generalised linear mixed model (see “Methods” for details). Interestingly, at per-gene level, we did not see any significant transcriptional changes in CD4+ T-cells, and only three genes that were significantly differentially expressed in CD8+ T-cells (FDR < 10%, Supplementary Data 3A). Within B-cells, we identified 97 genes with significant changes with age (FDR < 10%, Supplementary Data 3B). Given that we observed an increase of genes expressed after 77 weeks of age we also tested for differences between cells from the three youngest ages and the oldest age. We found one gene in CD4+ T-cells (Supplementary Data 3C), and two genes, which were also found in the quantitative analysis, in CD8+ T-cells (Supplementary Data 3D). Leveraging the Tabula Muris Senis (TMS) dataset, we replicated these associations and found that 37 out of the 86 B-cell associations replicated (same effect direction and nominal *p* < 0.05, 11 genes were not tested in TMS; see “Methods” for details). The CD8+ T-cell ageing genes could not be replicated in the TMS dataset. Within the B-cells, we found an additional 57 genes that were exclusively significantly differentially expressed between the three youngest ages and 101 weeks (Supplementary Data 3E). Most of these genes (30) were in the positive direction in the discrete part of the MAST model, matching our prior observation that a larger number of genes are expressed in older ages. Sixty-six of the 97 global age-associated B-cell genes increased in expression level with age, and among this set, we found a strong enrichment for ribosomal RNA processes (Supplementary Data 3D, Supplementary Data 4). This is in line with previous studies in blood transcriptomic changes (Frenk and Houseley^23^; Teo et al.^19^). Lastly, we assessed age-related DNAme changes in both enhancers and promoters, and identified 3 enhancers, all in CD4+ T-cells, and 48 promoter regions, 22 in CD4+ T-cells and 27 in CD8+ T-cells, that had age-associated changes (Supplementary Data 5).

### Modelling epigenetic ageing in single cells

We next set out to quantify the difference in epigenetic ages within and between the different time points. To do so, we built a DNAme ageing clock (scEpiAge) to predict the ages of individual cells. In contrast to classical epigenetic ageing models, we leveraged an approach that is flexible enough to overcome differences in coverage and can deal with a high degree of sparsity, thereby making it applicable to single cell as well as sparse bulk data. Specifically, we built models trained on blood samples and liver samples to predict epigenetic age in these tissues. We combined newly generated bulk DNAme data (*n* = 226), which included sorted immune cell types (*n* = 32) and liver samples (*n* = 33), [new Babraham dataset; see “Methods” for details] with five publicly available bulk datasets^4,5,24–26^, totalling 411 samples (Supplementary Data 6, 262 in blood and 138 in liver) and age ranges from 1 to 153 weeks (3 to 153 weeks for blood, and 1 to 128 weeks of age for liver).

**Fig. 2:**
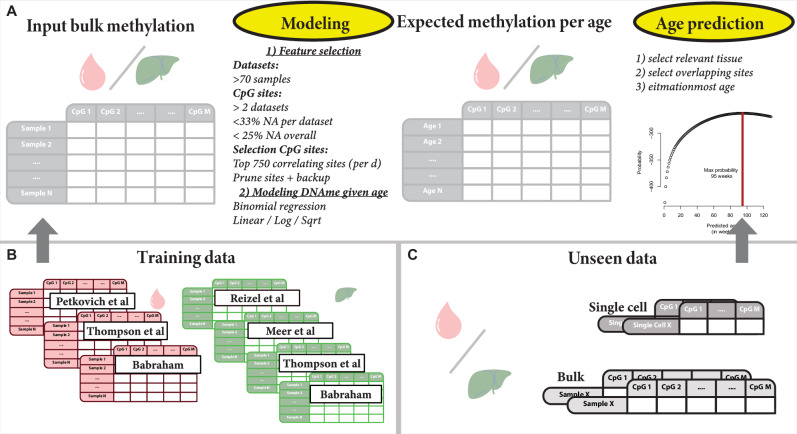
Overview of the scEpiAge modelling setup. **A**–**C** Schematic representation of the scEpiAge model during training, testing and application. **A** Modelling setup going from input data (left) via feature selection and age modelling (left centre), to an expected DNAme given age matrix (right centre) and to application for age prediction (right). **B** Datasets used to train the two scEpiAge models: We selected DNAme datasets from blood and liver, which we generated in this study or obtained from five published studies^4,5,24–26^. **C** Depiction of unseen data either in the validation stage or after model building. Created with BioRender.com released under a Creative Commons Attribution-NonCommercial-NoDerivs 4.0 International license.

The scEpiAge setup, illustrated in Fig. 2, is similar to the genetic and epigenetic distance-based prediction methods used in other settings^27–30^ and also by another recent approach to predict the epigenetic age of single cells^17^. Our method includes the following modifications over the currently published methods: (1) we generalised and validated it to work on both (sparse) bulk and single-cell data, and (2) we optimised the feature selection and modelling (see “Methods” for details). In addition, we used a significantly larger number of reference samples (2.3 times more samples for liver and 3.6 times more samples for blood (Fig. 2B)) originating from five different studies.

**Fig. 3:**
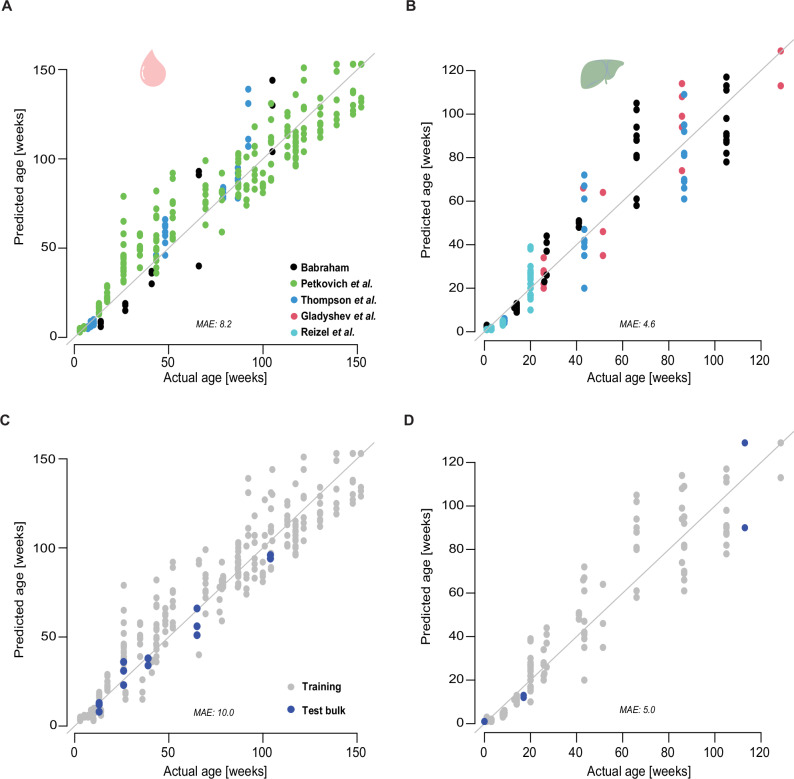
DNAme-based age prediction performance in bulk. **A**–**D** Performance of the model on training data and independent validation data, performances are shown in median absolute error (MAE). Performance of the models on the training set in blood (**A**) and liver (**B**) (colours represent the different datasets); performance of the models on independent bulk blood (**C**) and liver (**D**) data, the colours represent training (grey) or test (blue) label. Created with BioRender.com released under a Creative Commons Attribution-NonCommercial-NoDerivs 4.0 International license.

The best scEpiAge model has a cross-validation median absolute error (MAE) of 8.2 weeks of age in blood and a MAE of 3.9 weeks in liver. When applying the model to the whole bulk training dataset we obtain a final MAE of 8.2 in blood and 4.6 in liver (Fig. 3A, B). Using left-out validation samples, we could validate these errors, which we found to be in line with the error on unobserved data (Fig. 3C, D; MAE: 5 for liver and MAE: 10 for blood).

To gain insight into what types of sites are important for the scEpiAge models we performed site enrichments, including the backup sites, as well as for the Stubbs et al. multi-tissue clock sites^5^ (Supplementary Fig. 4). We find that the enrichments for the main scEpiAge models, without backup sites, and the models including backup sites have the same significant enrichment regions, and only marginally different enrichment statistics. There is only one class of features, coding sequences in the blood specific scEpiAge model, that is not significantly enriched when not taking into account the backup sites, but the enrichment statistic is very similar in both settings. This shows that the putative biologically relevant effects captured by the main and backup sites are highly complementary. In general, we find that the scEpiAge-liver model selects similar regions as compared to the Stubbs et al. multi-tissue model while the blood model selects markedly different regions. In the scEpiAge-blood model, we do not see the significant depletions for promoters, CGIs and 5’ UTR regions that we do observe in both the scEpiAge-liver model and the Stubbs et al. multi-tissue model^5^. It is worth noting that the Stubbs et al. multi-tissue model is built based on heart, lung, brain, and liver DNAme datasets, but does not include blood DNAme data. This could explain the discrepancy in site enrichments between the various modes.

Next, we compared scEpiAge to a classical regression-based epigenetic ageing model. We decided to use the Stubbs et al. clock^5^ as an example of this type of model and compared it to our scEpiAge-liver model. For the comparison, we only considered samples in the range of 1–41 weeks of age, to match the published model. We found that the original Stubbs et al. model (including normalisation) only works on samples from the original cohort^5^ and the Reizel et al. study^25^, both samples that comprise the training and testing data of the Stubbs et al. clock. All other samples, i.e., Meer et al.^24^ or Thompson et al.^26^, do not generate a prediction. Importantly, the scEpiAge model can predict ages for samples from all studies. We then focused on the samples (*n* = 63) that were part of the training sets in both models. The MAE for these samples in the Stubbs et al. model is 0.8 weeks and 3 weeks in the scEpiAge-liver model. The correlation between the predictions of the two models is high (Pearson *r*^2^ = 0.87), but lower than each one individually to chronological age (Pearson *r*^2^: 0.92 for Stubbs et al. model; and Pearson *r*^2^ = 0.88 for scEpiAge-liver).

Given that scEpiAge can predict ages from sparse bulk datasets, we decided to look at publicly available DNAme studies focusing on various interventions (drugs, diet, genetic models) which were shown to affect health or lifespan in mice. We found that scEpiAge was compatible with all included data types, i.e., with reduced representation bisulfite sequencing (RRBS) as well as whole-genome bisulfite sequencing (WGBS) datasets, and was able to detect differences in predicted epigenetic age related to various interventions. Predicted epigenetic age was affected by the dietary fat content (Supplementary Fig. 5A)^31^, and also by caloric restriction but not by rapamycin treatment in 22-month-old mice (Supplementary Fig. 5B)^32^. Long-lived Ames Dwarf mice, which are known to epigenetically age slowly, showed a large reduction of predicted epigenetic age using our model (Supplementary Fig. 5C)^32^. Lastly, genetic knockout of methionine adenosyltransferase 1a in the liver, which causes the spontaneous development of steatohepatitis, increased the predicted epigenetic age in 10-month-old mice (Supplementary Fig. 5D)^33^.

**Fig. 4:**
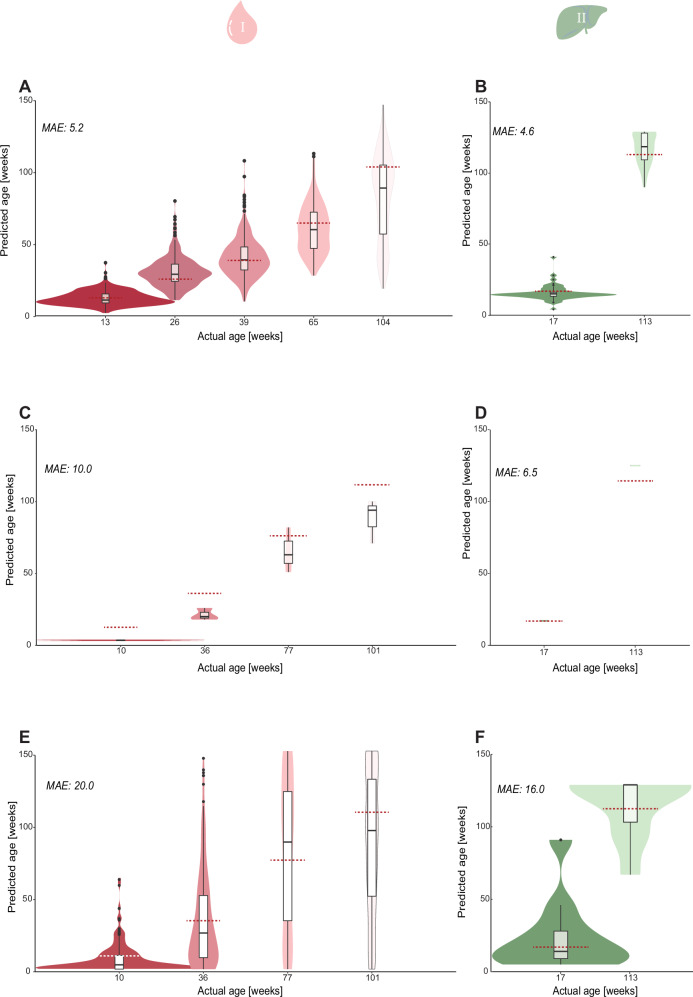
Predicting methylation age in simulated and real single-cell data. The predictions for epigenetic age shown in violin and boxplots in this figure have been made using the scEpiAge model for blood or liver described in Fig. 2, performances are shown in median absolute error (MAE). Boxplots show median levels and the first and third quartile, whiskers show 1.5× the interquartile range. **A**, **B** Predicted DNAme age of simulated blood (**A**) or liver (**B**) single cells. **C**, **D** Predicted DNAme age of pseudo-bulked real blood (**C**) and liver (**D**) single-cell data. **E**, **F** Predicted DNAme of the single-cell blood (**E**) and liver (**F**) data. The blood data is shown in red, and liver in green, the shading represents age (dark young, bright old). Created with BioRender.com released under a Creative Commons Attribution-NonCommercial-NoDerivs 4.0 International license.

To assess the performance of the model on single cells, we simulated 50 single-cell methylomes, by downsampling bulk samples in the validation dataset (see “Methods”). We found that the error on the simulated liver cells was 4.6 weeks of age, and in blood, the error on the simulated single cells was 5.2 weeks of age (Fig. 4A, B). Next, we moved to predicting age in the newly generated single-cell data, using our blood dataset together with published single-cell methylation data from mouse hepatocytes^13^. First, we assessed the performance at pseudo bulk level, aggregating the cells by donor, to estimate the impact of batch effects and different sequencing methods. We found in blood a MAE of 10 weeks and in liver a MAE of 6.5 weeks, both in line with the errors in bulk (Fig. 4C, D). Observing close-to-expected age predictions in the pseudo-bulk setting, we moved to actual single-cell predictions, where we found that the error of the scEpiAge model increased substantially. We can predict ages in all cells which pass QC, both in blood and liver, with the MAE increasing to 26 weeks in blood and 16 weeks in liver, and expected and real age are correlated (Pearson *r* = 0.6; *p* < 2.2e-16). We observed a relationship between the age prediction and the coverage of the cells in blood (*r* = −0.36, *p* < 2.2e-16), indicating that the number of covered sites influences our ability to accurately predict age. Based on simulation, we noted that with at least 40 covered sites, the mean error is below 1 week of age for the simulated cells (see “Methods”, Supplementary Fig. 6). We therefore included this requirement, and could retain 72.5% of all cells in our single-cell blood data (95% of cells in the liver data). The MAE of the scEpiAge predictions on single cells decreased to 20 weeks in blood and 16 weeks in liver (Fig. 4E, F).

Other groups have also generated models to predict epigenetic age based on DNA methylation at single-cell resolution, notably the study by Trapp et al.^17^. We therefore set out to compare the two modelling approaches. We generated a reimplementation of scAge^17^ on the same training set as used for scEpiAge and found that the cross-validation MAE of scAge on bulk samples is 16 weeks in blood, and 7 weeks in liver (see “Methods”; Supplementary Fig. 7), while scEpiAge has a MAE of 8.2 weeks in blood and a MAE 3.9 weeks in liver (Fig. 3A, B), indicating the improvements of our model and extended dataset.

Next, we assessed the performance of the published scAge^17^ on 50 simulated single-cell methylomes (see above). The MAE of scAge on the simulated liver cells was 8.3 weeks (compared to 4.6 weeks for scEpiAge) and on the simulated blood cells 10.0 weeks (compared to 5.2 weeks for scEpiAge) (Supplementary Fig. 8A, B and Fig. 4A, B). Finally, we compared the performance of scAge to scEpiAge on actual single-cell data using our single-cell blood dataset and published single-cell methylation data from mouse hepatocytes^13^. Using the Trapp et al. model on the blood data, the MAE of scAge is 25.98 weeks, and of scEpiAge, the MAE is 20 weeks. When assessing performance in the same way on the hepatocytes, the MAE of scAge is 19.8 weeks versus 15.5 weeks for scEpiAge (Supplementary Fig. 8E, F and Fig. 4E, F). The Spearman correlation between the scEpiAge and scAge predictions in blood is 0.64 (Spearman rho, Supplementary Fig. 9A), while in liver, the correlation is 0.75 (Spearman rho, Supplementary Fig. 9B). Overall, this shows that the published scAge model^17^ can predict epigenetic age in blood and liver, but the modelling changes of scEpiAge, as well as the inclusion of an extended dataset, improve the prediction accuracy.

### Epigenetic age predictions in blood at single-cell resolution

**Fig. 5:**
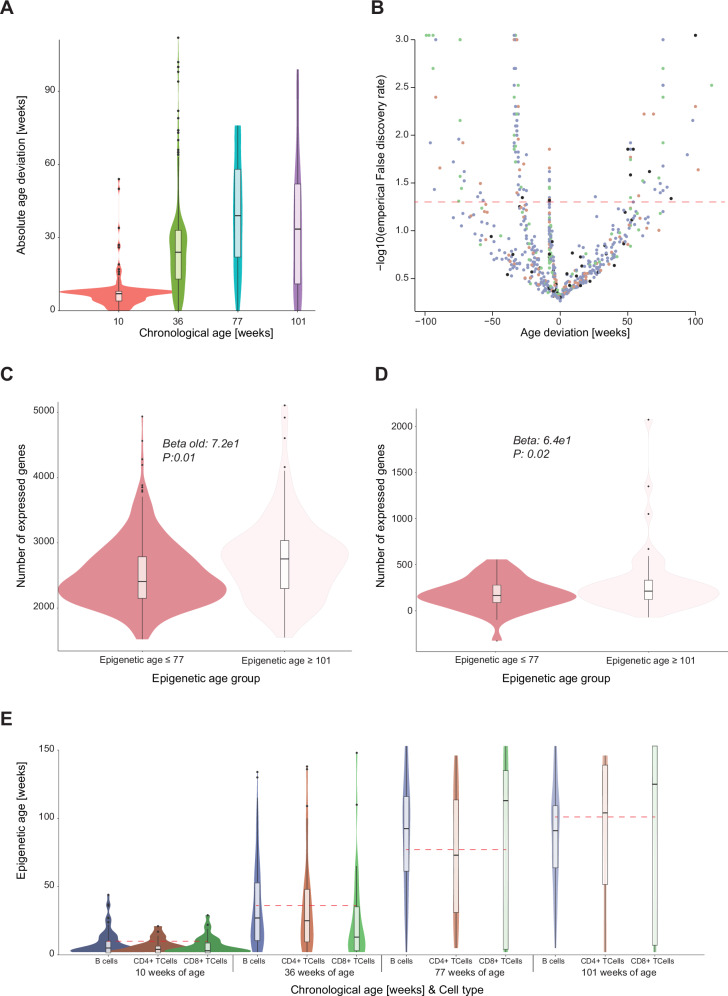
Epigenetic age predictions in blood at single-cell resolution. **A** We calculated the deviation between the real chronological age and the DNAme age predictions of our scEpiAge model for each single cell. Depicted are the deviations for each age group in violin plots and boxplots. The colours represent the different ages (matched to Fig. 1D). **B** For each cell, we estimated the empirical false discovery rate and plotted these against the age deviations between chronological and DNAme age. Individual cells are coloured by cell type: blue B-cell, orange CD4+ T-cell, green CD8+ T-cell, black other. **C**, **D** Number of expressed genes in cells with a DNAme age below 77 weeks or DNAme age above 101 weeks, showing cells from **C** all chronological age groups or **D** only from chronological age 101 weeks. The shade of red indicates the age groups of the cells. **E** Epigenetic age predictions per major cell type, grouped by chronological age (blue B-cell, orange CD4+ T-cell, green CD8+ T-cell, the shading represents age (darker younger, brighter older)). The boxplots in **A**, **C**–**E** show median levels and the first and third quartile, whiskers show 1.5× the interquartile range. Statistics shown in 5c&d from a linear model testing the number of expressed genes over 101 weeks or under, when correcting for read depth and cell type.

Next, we focused on the data generated from blood cells spanning a wide range of ages and predicted DNAme age for each cell (Fig. 4E). When taking a closer look at these predictions, we observed that the median scEpiAge predictions of cells at a given age were very similar to the expected age, i.e., at 10 weeks of age the mean prediction was 8.4, at 36 weeks of age it was 36.2, at 77 weeks of age it was 83 and at 101 weeks of age it was 88. The variance increases with age, with a standard deviation of 10.2 at 10 weeks, 32.7 at 36 weeks, 45 at 77 weeks, and 42 at 101 weeks. We asked if the observed epigenetic age predictions and the individual cellular deviation from the organismal (chronological) age reflect potential biological differences or alternatively result from possible technical noise of the model. We leveraged cell-specific simulation, based on the expected age and CpG sites covered in the cell of interest, to find outliers (see “Methods”). We found a total of 120 cells (19.4%) with a significant deviation from the expected age (Fig. 5A; empirical FDR 5%), i.e., cells where the predicted epigenetic age deviated significantly more from the expected (chronological) age than technically expected. Most of the significant deviations were cells that were significantly younger than expected (84 out of 120). This is in line with the error that shows more underpredictions as compared to overpredictions (Fig. 5A). Most of the extreme predictions were found at 36 weeks of age, where 24% of the predictions significantly deviated from the expected age, of which 20% were predicted to be younger and 4% predicted to be older.

We then asked if the scEpiAge differences are associated with global and specific gene expression effects. As observed for chronological age, we observed a significant increase (LM *p* = 0.1) in the number of expressed genes in older cells (epigenetic age ≥ 101 weeks) versus younger cells (epigenetic age ≤ 77 weeks) (Fig. 5B). Given the relation between epigenetic age and chronological age we also tested this within each chronological age, and corrected the number of expressed genes for the number of sequenced reads, where we could also significantly identify this effect in the 101-week-old cells (LRT *p* = 0.02) (Fig. 5C). In other chronological age groups we could not replicate the effect; however, the number of old cells (epigenetic age ≥ 101 weeks) in these categories is low. When replicating effects from chronological age, we observe that we can replicate three of the 97 ageing-associated genes in B-cells (Supplementary Data 3B). In a subsequent genome-wide analysis, we did not find any significant changes in B-cells or CD4+ T-cells. In the CD8+ T-cells, we identified 3 genes that were differentially expressed with ageing (FDR < 10%), i.e., *Ttn*, *Rpl36a* and *Txnip* (Supplementary Data 3F).

The use of the scM&T-seq^18^ protocol provides the unique opportunity to build multimodal clocks; however, given the overall small number of donor animals we could not build a clock leveraging individual genes as a predictor. As such, we did assess the impact of adding the number of expressed genes as an extra predictor and built a staged model leveraging both the scEpiAge information and number of expressed genes as predictors. We find that the model significantly improves the MAE of the prediction of the individual cells (MAE scEpiAge 20 vs 19 in the multimodal model). Interestingly, while the overall MAE decreases, the MAE in the 10-week-old cells increases (Supplementary Fig. 10).

To try and link scEpiAge to other biological ageing effects, we linked the number of somatic mutations, which individuals accumulate over their lifespan^34–36^. We tested both the relation of the scEpiAge prediction with the number of mutations on the whole genome as well as a focused analysis on the mitochondria. We find a significant positive relation between the number of sites with alternative alleles, a proxy for the number of somatic mutations, and the epigenetic age as calculated per cell type (MT: *p* = 5 × 10^−8^, genome-wide: 7.03 × 10^−24^, “Methods”), strengthening the link between biological ageing and scEpiAge.

Interestingly, when splitting the scEpiAge predictions per major cell types, i.e., B-cells, CD4+ and CD8+ T-cells, and others, we observed that the predictions for CD4+ and CD8+ T-cells were, in general, lower as compared to B-cells. On average, CD8+ T-cells are estimated to be 3.5 weeks younger than B-cells, CD4+ T-cells are found to be 0.5 weeks younger than B-cells. Of note, the model overall underestimates epigenetic age by 1.3 weeks compared to chronological age (Fig. 5D). We also observed that a large number of T-cells had significantly younger age predictions (25.3% of CD8+ T-cells and 15.6% of CD4+ T-cells, versus 11.6% of B-cells), but T-cells also had more extreme age predictions on the other hand, with 8.4% of the CD8+ T-cells estimated to be significantly older than expected (5.8% of the CD4+ T-cells), versus only 4% of the B-cells. To verify these findings, we investigated age predictions of sorted immune cells, i.e., CD4+ T-cells, CD8+ T-cells, and B-cells, and observed that in bulk, CD4+ and CD8+ T-cell data ages were in all but one case lower as compared to the B-cells from the same mouse (Supplementary Fig. 11), which is in line with our single-cell data.

## Discussion

Ageing at the organismal or tissue level is characterised by progressive functional decline and deterioration, increasing the risk of disease and death^37,38^. Unicellular organisms show signs of ageing and are generally not immortal^39^, however our knowledge of how individual cells age within multicellular organisms remains poorly understood. Several scenarios are possible to explain the age-related changes of tissues, of which the extremes would be that (1) all cells age at a uniform rate and homogeneously lose their functional capacity, or (2) cells only have 2 possible age-states, i.e., young and old, and the age of a tissue is determined by the relative proportion of young and old cells. Ageing at the single-cell level could also be a combination of these two extreme scenarios, where cells age at different rates and functional decline occurs at varying rates. Addressing these fundamental questions has remained challenging and despite several studies assessing transcription in single cells at varying ages^21,40^, we cannot yet resolve this issue. The use of epigenetic, in particular DNA methylation-based, age predictions has thus far shown to be the most accurate way to assess biological age^2,3,5^ and this motivated us to develop two scDNAme age predictors (scEpiAge for blood and liver) to explore this concept in single cells and generate a large single-cell multi-omics dataset, assessing DNA methylation and transcription from nucleated blood cells from mice spanning a broad range of ages.

The development of a single-cell age predictor was necessary, as single-cell data differs in key aspects from bulk sequencing data, being very sparse and primarily binary. Our epigenetic ageing models (for liver and blood) address these challenges by building on bulk methylation datasets (newly generated ones and publicly available ones), from corresponding tissues, to generate a theoretical methylation profile for each age (and tissue type). Distinct from previous generations of epigenetic clocks^2,5,9,24,26^, the scEpiAge model can be applied even when not all sites are present, lowering the accuracy but enhancing usability substantially. To further improve performance, we included the use of backup sites, CpGs with a DNAme profile similar to and in close proximity to the main site. Additionally, we show that our generalisations in the modelling of the expected DNAme profiles increase accuracy over multiple datasets and in complex tissues such as blood, relative to a recent similar clock model by Trapp et al.^17^. By leveraging in silico simulated single-cell DNAme profiles matching the sites covered in a single cell and the DNAme profile at a given age, we can now add a confidence limit on how certain we are that a predicted age is out of the expected range. Lastly, our model can be applied to (sparse) bulk bisulfite data, to low-depth long-read sequencing methods and also to large-scale screening approaches, enhancing the usability of epigenetic age models on sequencing data in general.

When initially exploring our mouse blood single-cell multi-omics dataset spanning a broad range of ages, we found only minor age-related differences. Cell type proportions only showed a small but non-significant overall increase of B-cells with age (Fig. 1B), in line with prior reports^19^, and similarly, we only observed minor transcriptional changes of the single cells at specific cell type level (Fig. 1D). Nonetheless, several of these differentially regulated genes had previously already been associated with ageing^19^, suggesting that some of the transcriptional changes do reflect age-related effects. Interestingly, we found a strong effect on the number of genes expressed with age, which we replicated in external datasets. The number of genes expressed first decreases minimally up to 77 weeks of age in our data, followed by a strong increase at our 101-week-old time point. We designed our replication analyses to match this phenomenon and found that it replicates in several Tabula muris tissues and in a large-scale human PBMC dataset. To better dissect this phenomenon in the future a more dense sampling procedure will be required as well as more replicates. Notably, adding the number of expressed genes to scEpiAge, i.e., the staged model, resulted in an improved prediction of chronological age (Supplementary Fig. 10). This will be useful in, but is also limited to, studies where combined single-cell data is available.

Interestingly, we found that DNAme levels at CGIs increased with age in the single-cell dataset, and in repeat regions, we observed a weak but significant negative correlation with age (Fig. 1F), and both results could be confirmed in bulk data from sorted immune cells. This result indicates that cell type-specific global changes might be accumulated over time but have likely been masked due to cellular heterogeneity in past studies. These DNAme changes could be the result of imperfect DNAme maintenance, resulting in loss of DNAme at hypermethylated (repeat) regions, and of aberrant de novo methylation resulting in gain of DNAme at hypomethylated (CGIs)^41,42^.

It is worth noting that due to the technological limitations of single-cell methylation methods, the number of cells we profiled is relatively large compared with published DNAme datasets yet small compared with single-cell RNA-seq datasets. As such, our transcriptional analyses are relatively low-powered, and we may not be able to detect smaller age-related transcriptional differences in our study, and in particular small transcriptional effects caused by small DNAme changes in CGI promoters or repeats. Additionally, it is worth noting that DNAme datasets are inherently sparse, which negatively impacts the power of our analyses.

Comparing the predicted ages with the known chronological ages, we found that both pseudo-bulk datasets, as well as the average age of all cells of a given age group, agreed with the expected (chronological) age, showing the consistency of our approach and prior models^5,17^. A comparison of our scEpiAge-liver and scEpiAge-blood predictor showed that epigenetic ageing signals are stronger in the liver as compared to blood, possibly reflecting the more complex cell type distributions in blood. It should also be noted that while we had more training data for the scEpiAge-blood model (than for scEpiAge-liver), these were primarily heterogeneous and not cell type-specific (FACS sorted) datasets, which made identifying a global, rather than cell type proportion affected age signal, more difficult and potentially leading to a less robust model. Alternatively, the differences could be explained by dataset and age span differences, where the scEpiAge-blood model works across a larger window and the data is less uniformly distributed over data sources and ages, which might negatively impact the modelling. We assume that these reasons combined explain that even with fewer samples in the liver (138 liver versus 262 blood), we were able to predict ageing in the liver more precisely as compared to blood (MAE 3.9 in liver versus 8.2 in blood).

A closer analysis of the single-cell scEpiAge predictions uncovered ageing heterogeneity, which was larger than technically expected for 19.4% of the cells. This indicates that according to scEpiAge, younger and older cells co-exist and strikingly, even at older ages, a pool of epigenetically young cells remains, in line with a recent in vitro study, which shows that the epigenetic age heterogeneity observed between cell clones from the same donor is greater than the variability of the clock prediction^43^. In terms of transcriptional differences with age, interestingly, we found differential expression of *Rpl36a*, a ribosomal gene which is down-regulated with higher scEpiAge, which is in line with our results on chronological age and the literature^23^. Also, *Txnip*, a major regulator of cellular redox signalling which protects cells from oxidative stress, was found to be significantly lower expressed at higher scEpiAges, and the loss of *Txnip* was shown to induce premature ageing in hematopoietic stem cells^44^. Follow-up work will increase the sample number and, hopefully, thereby increase the statistical power to detect other relevant putative functional differences.

The fact that we also found most of the extreme predictions at 36 weeks of age, where 24% deviated from the expected age, (20% predicted to be younger and 4% predicted to be older), could suggest differences in ticking rates between cells at a given age, most pronounced in mature mice. Furthermore, our results showing that CD4+ and CD8+ T-cells were consistently younger when compared to B-cells from the same mouse (Supplementary Fig. 11), could indicate that T- and B-cell ageing differs. These findings need to be verified in a larger dataset, preferably using predictive models trained separately for each blood lineage.

In summary, we have developed a framework to expand our understanding of ageing, to address key questions related to cell type-specific age-related changes and to better understand the process of rejuvenation in single cells. The future applications are manifold and importantly, our epigenetic age prediction concept can also be applied to other species, including humans.

## Methods

### Animals and sample collection

This research complies with all relevant ethical regulations. Animal experiments were performed according to the UK Animals (Scientific Procedures) Act 1986, license PPL 70/8303 and approved by the Babraham Institute Animal Welfare and Ethics Review Body.

Mice were bred and maintained in the Babraham Institute Biological Services Unit (BI BSU) under Specific Opportunistic Pathogen Free (SOPF) conditions. C57BL/6 J mice (supplied by Charles River Laboratories) were imported into the BI BSU by embryo transfer and bred there (/Babr) as a SOPF colony in plastic film isolators. After weaning, male mice were transferred to individually ventilated cages in groups of between two and five mice. All mice were fed CRM (P) VP diet (Special Diet Services) ad libitum and received sunflower seeds, poppy seeds or millet at cage-cleaning as part of their environmental enrichment. The health of mice was monitored closely, and any mouse exhibiting clinical signs of ill-health or distress that persisted for more than 3 days was culled. In this way, all the mice maintained, remained sub-threshold with respect to the UK Home Office severity categorisation. Any mice exhibiting any gross pathology upon post-mortem examination were excluded from this study.

Samples for bulk datasets were collected as described before (Stubbs et al.). All tissues were snap-frozen directly after isolation. Genomic DNA was isolated from ~10 mg frozen tissue using the DNeasy Blood & Tissue Kit (Qiagen #69504).

Sorted immune cell types (CD4+, CD8+ T-cells, and B220+ B-cells) were collected from the same animals used in the Stubbs et al. study^5^. Briefly, samples were incubated with anti-mouse TCR β chain (clone H57-597; Biolegend #109207), anti-mouse CD4 (clone GK1.5; Biolegend #100449), anti-mouse CD8a (clone 53-6.7; Biolegend #100759) and anti-mouse B220 (clone RA3-6B2; Biolegend #103211). All antibodies were used at a dilution of 1:300. Cells were then washed once in PBS with 0.5% BSA, and flow-sorted (BD FACSAria III cell sorter) directly into RLT plus lysis buffer (Qiagen #1053393). Lymphocytes were gated based on forward and side scatter, then gated on singlets. T-cells were selected by gating for TCRβ+ and then gating for CD4+ or CD8a+. B-cells were selected by gating for TCRβ- cells and then gated for B220+ cells (Supplementary Fig. 13a).

Blood samples for single-cell analysis were collected and directly processed. Red blood cells were lysed using 2 rounds of red blood cell lysis (RBC Lysis Buffer, Roche #11814389001), followed by 3 rounds of washes with PBS with 0.5% BSA. Cells were then stained with Hoechst 33342 (ThermoFisher Scientific #H3570) and flow-sorted (Supplementary Fig. 13b) into 96-well plates containing 2.5ul of RLT plus lysis buffer (Qiagen #1053393). Cells were gated on Hoechst+, FSC-A vs SSC-A, and then FSC-A vs FSC-H to ensure nucleated single cells were deposited in each well. Plates were then frozen and stored at −80 °C until processing.

### scM&T-seq library preparation

96-well plates were thawed on ice and processed as follows. First, mRNA was captured on oligo-dT conjugated streptavidin magnetic beads (MyOne C1, ThermoFisher #65001) and physically separated from single-cell lysates prior to reverse transcription using Superscript II (ThermoFisher #18064014) and PCR amplification using KAPA Hifi (Roche 07958935001). Illumina-compatible libraries were prepared from the amplified cDNA using the Nextera XT kit (Illumina Roche #07958935001). Genomic DNA was purified from lysates using AMPure XP beads (Beckman Coulter #A63880) and then bisulfite-converted using the EZ-96 DNA Methylation-Direct MagPrep kit (Zymo, D5044) after which single-cell bisulfite sequencing (scBS-seq) libraries were prepared using random primed first and second strand synthesis with Klenow exo- polymerase (Qiagen P7010-HC-L) and PCR with KAPA HiFi (Roche #07958935001). Full step-by-step protocols have been published for both the separation and RNA-seq^45^ and scBS-seq method^46^. scRNA-seq libraries were sequenced on Illumina Hiseq4000 instruments using 75 bp paired-end reads and pooling 384 cells per lane. scBS-seq libraries were sequenced using 150 bp paired-end reads pooling 48 cells per lane.

### Bulk and sorted immune cell types RRBS library preparation

RRBS libraries were prepared from isolated DNA as described before^5^. Briefly, RRBS libraries were prepared by MspI digestion of 100–500 ng genomic DNA, followed by end-repair and T-tailing using Klenow Exo- (Qiagen P7010-HC-L). Illumina-compatible adaptor ligation was performed overnight using T4 DNA Ligase (NEB #M0202), followed by a cleanup step using AMPure XP beads (Beckman Coulter #A63880, 0.9x). Subsequently, libraries were bisulfite treated using the EZ-96 DNA Methylation-Direct MagPrep kit (Zymo, D5044). The libraries were amplified using KAPA HiFi Uracil HotStart DNA Polymerase (Roche #07959079001). All amplified libraries were purified (AMPure XP beads, Beckman Coulter #A63880, 0.8x) and assessed for quality and quantity using High-Sensitivity DNA chips (Agilent #5067-4626) on a Bioanalyzer (Agilent). High-throughput sequencing of all libraries was carried out with a 75 bp paired-end protocol on a HiSeq 2000 instrument (Illumina). For this project, we extended the data generated for the Stubbs et al. study by: (1) generating extra RRBS samples coming from older mice but the same tissues (Liver, Lung, Cortex and Heart), extending the age range of the Babraham study to 105 weeks maximum, and (2) additional RRBS runs were generated on new tissues and cell types, ages ranging from 14 weeks to 105 weeks. This second extension contains sorted immune cells leveraged in this study (B-cells, CD4+ T-cells, and CD8+ T-cells), but also data on cerebellum, hind muscle, kidney, testes as well as intestinal stem cells, and lung macrophages.

### External datasets

In addition to the newly generated data, the following publicly available datasets were downloaded from GEO, processed as described below and included in the analysis:

GSE93957^5^, GSE80672^4^, GSE60012^25^, GSE121141^24^, and GSE120137^26^, SRA344045^13^.

### scM&T-seq data processing

scM&T-seq data was processed as described previously (DNAme^18^; RNA^47^). scBS-seq data was processed as described previously; briefly, reads had 6 bp removed on their 5’-ends to remove the random primed portion of the reads, and were also adaptor- and quality-trimmed using Trim Galore (v0.6.7; options --clip_r1 6). Trimmed reads were aligned to the bisulfite-converted GRCm38 mouse genome using Bismark v0.22.3 in single-end mode (options: --non_directional)^48^. Methylation calls were extracted after duplicate reads had been removed (deduplicate_bismark). scRNA-seq data was processed as described previously, briefly: the RNA-seq reads were adaptor and quality-trimmed using Trim Galore (v0.6.7), and aligned to the GRCm38 mouse genome build using STAR (2.7.1a)^49^, in two-pass mode. Expression quantification was performed leveraging FeatureCounts available in Subread 1.6^50,51^, and based on ENSEMBL version 96^52^.

### Single-cell quality control and normalisation

scM&T-seq data was quality controlled pr level. On the DNAme side, we removed cells with a low read depth (removing cells with <1 M reads), removed cells with a low unique number of mapping reads (cells with <50,000 uniquely aligned reads are removed), and high non-CpG methylation levels (cells with non-CpG meth >20% are removed). This left a total of 853 high-quality cells. For downstream analysis, we mapped all CpGs to the forward strand and removed non-CpG or ambiguous methylation calls.

Quality control of scRNA-seq data was performed using the SCATER package^53^. Cells were retained for downstream analysis if they had at least 150,000 counts from endogenous genes, at least 1500 genes with non-zero expression, less than 90% counts came from the top 100 highest expressed features and less than 15% mitochondrial reads. After quality control, 981 out of 1055 blood single cells were considered for downstream analysis. For further analyses, expression counts were SCRAN normalised into counts per million and log transformed (log(normCPM+1)).

During our analysis, we found that a proportion of cells had very high expression values for haemoglobin genes (Hbb-bt, Hbb-bs, Hba-a1, and Hba-a2); leveraging the expression counts of these genes, we estimated the number of red blood (RB) cells contaminating our expression level. The expression of the four genes formed distinctive peaks, around no expression, around 5 reads per gene per cell and around 10 reads per gene per cell. We combined the information over the marker genes and defined cells with on average 5 reads per marker per cell as having 1 RB cell as contamination (348 cells), and if on average more than 10 reads per marker per cell was found we defined it as 2 contaminating RB cells (69).

### Cell type annotation

We leveraged a combined de novo and reference-based mapping setup to annotate cells to cell types. Specifically, for the de novo, we leveraged shared-nearest neighbour (SNN) from Seurat, as input we used the first 10 PCs, derived from the top 2000 highly varying genes that are expressed in at least 1% of the cells, and the SNN clustering resolution was set to 0.5. This yielded 12 clusters. In parallel we performed a reference-based cell annotation, to do so we took the bulk RNA-seq data from the haemopedia resource^54^. The resource contains Bulk RNA-seq data of 57 different flow-sorted mouse blood cell types, originating from 13 different cell lineages and are derived from healthy mice. The raw data was reprocessed with the same pipeline as our single-cell data, see above. From this, we simulated single cells leveraging Splatter^55^, expression counts were taken from the bulk data, and other parameters needed for the simulation were derived from the actual sc-RNAseq data. By leveraging SingleR^56^ we subsequently annotated the individual cells to the best matching simulated single cells from Heamopedia and annotated them to the relevant cell type. Lastly, we combine the information from SNN and SingleR and do a majority vote per cluster to assign final cell types per cluster and thereby cells. We leveraged the cell type annotation derived from RNA also in the DNAme analyses. Given the low cell numbers, we focused the cell type-specific analysis on B-cells, CD4+ T-cells (EffCD4T, MemCD4T, NveCD4T, RegT), and CD8+ T-cells (MemCD8T, NveCd8T).

### Bulk RRBS data processing and normalisation

RRBS and WGBS datasets were processed as described previously^5,57^, aligning reads to the bisulfite-converted GRCm38 mouse genome using Bismark v0.22.3^48^. After mapping we transformed the DNAme calls to the forward direction and removed non-CpG or ambiguous methylation calls.

We performed per tissue (liver and blood) and dataset (Petkovich; Reizel; Meer; Thompson; Gravina; Babraham_p1, Babraham_p2, Babraham_p3) quality control. To do so, we selected sites that had at least 5X coverage in 80% of the samples, we dropped samples with high missingness rate (>25%) and subsetted per dataset tissue combination to sites present in all samples. Based on this set we did a PCA, again per dataset tissue combination, and dropped outliers on PC1 and PC2, we defined outliers as samples with scores higher (or lower) than mean plus (or minus) two times SD on each of the two PCs.

### UMAP on expression and DNAme

To get an overview of the global structures in both the gene expression data and the DNAme data, we used UMAP^58^. On expression, we used the top 5000 most highly variable genes and used the runUMAP function from the SCATER^53^ package with parameters n_neighbors=8, min_dist=1 to make the UMAP plots (Fig. 1C, D). On DNAme we joined the information on DNAme levels on promoters and enhancers, given the sparsity we removed regions with observations in less than 80% of the cells. Next, we used the imputePCA() function from the missMDA^59^ package to impute the missing information. Subsequently, we selected the 25% most variable regions and made a UMAP on the 15 first PCs (Supplementary Fig. 1A, B).

### Tissue cell composition analysis

To test for effects of age on cell type composition we used propeller^20^, implemented in the Specle R package. We transformed the proportions using the logit function, leveraging getTranssformedProps() and used the propeller.annova() function to test for the effect of age and correct for multiple testing.

### Differential expression analysis

We performed differential gene expression analysis leveraging MAST in the three major cell types (B-cell, CD4+ and CD8+ T-cells). Age, or epigenetic age, was used as a continuous variable, and we treated number of expressed genes, mouse ID, and Haemoglobin contamination as covariates. For T-cell analysis we also took sub-cell type proportion into account. For each of the three test cell types, we filtered to: (1) protein-coding genes, (2) genes expressed in at least 25% of the cells, selected the genes with a high biological variation (by measuring the modelGeneVarByPoisson function implemented in SCRAN^60^ (var>1)), (3) and lastly only tested genes that are expressed in at least 5 cells in at least 2 distinct age groups. Additionally, we tested for a binary effect of ageing between the ages lower than 101 weeks versus the 101-week-old cells.

The MAST model implements a hurdle model that combines information from two tests, a continuous model (for cells with non-zero expression levels), and a binary model (testing expressed vs not expressed). The effect sizes of the continuous and discrete parts of the model are then combined to give one final output. We filtered these to not have significant results (nominal *p* < 0.05 but in opposite direction between the tests) and performed Storey’s Qvalue to account for multiple testing. Significance was defined at 10% FDR.

To replicate our age-associated genes, we leveraged the data from the Tabula Muris Senis project^21^. Given that there is no blood data, we selected all the tissues with CD4+ T-cells, CD8+ T-cells and B-cells and downloaded the preprocessed 10X data. We leveraged the same pipeline as above to test for age associations between expression and ageing. Given the multiple sources of the cells, we added a covariate to account for the tissue of origin and added a covariate for the sex of the mouse, as in the main model, we also correct for the number of expressed genes. Replication here was defined as same effect direction and nominal significance.

### Ageing affects numbers of genes expressed in mouse

To test the relation between age (and epigenetic age) and the number of genes expressed, we used a linear model, implemented in R. For the test in our ageing mouse blood data, we treated age as a binary variable (old: 101 weeks, young <101 weeks) and corrected for number of expressed genes, cell types, and Haemoglobin contamination unless otherwise specified.

For replication in the Tabula Muris data, we mimicked the binary analysis testing ages lower than or equal to 77 weeks versus ages higher than or equal to 101 weeks. Here again, we corrected for the number of sequenced reads, and, if appropriate, corrected for sex. For the replication in the human PBMC data we matched these age criteria by selecting individuals below or equal to 55 years as young (roughly matching the 77 weeks in mouse), and assigned individuals over 64 years of age as old (roughly matching the 101 weeks age in mouse). In oneK1K, we corrected the number of genes expressed for the number of sequenced reads, material batch, and sequencing pool.

### Differential methylation analysis

We performed the single-cell differential DNAme analysis leveraging a generalised linear mixed effect model (GLMM) implemented in lme4^61^ and R. We leveraged a binomial link function to capture the binary nature of single-cell DNAme data and leveraged the random effect to capture the effects driven by mouse In this setup we tested for differentially methylated regions (DMRs) in enhancers and promoters, in the three major cell classes (B, CD4+ T and CD8+ T-cells). We tested regions with DNAme information in more than 25% of the cells per cell type. The enhancer and promoter information was derived from UCSC.

To associate DNAme levels at CpG-island and repeat regions to ageing we combined all CpG sites in the relevant region category, covered in at least one cell of each of the donor mice. To assess the effect of age on the overall methylation levels, we used a Spearman rank test. To replicate this finding in the bulk data, we selected these same sites per category and tested for the same effect in the sorted immune cell data, again using a Spearman rank test.

### Modelling epigenetic age

To build an epigenetic clock that is able to deal with sparsity better as compared to standard (elastic net) regression models, we leverage a direct distance-based model similar to the Trapp et al. model and similar to genetic prediction models. We built these models based on the data of the Quality-0controlled RRBS samples as described above, the selection for CpGs is done independently from the QC procedure described above.

### Building the expected age–methylation matrix

The first step we took was to build up an expected methylation versus age matrix. To do so, we combined bulk RRBS blood (or liver) datasets, specifically we used new Babraham RRBS samples, GSE93957^5^, GSE80672^4^, GSE60012^25^, GSE121141^24^, and GSE120137^26^ and reprocessed the raw data as described above. Next, we select CpG sites that are covered in at least 2 studies, have at most 25% missingness per covered dataset, and 33% maximum overall missingness in the combined study. In the blood dataset we included the pseudo-bulked single-cell data to inform the site selection on non-RRBS data. This leaves 366,250 CpG sites in blood and 753,296 CpG sites in liver.

After this initial selection, we calculate per datasets and per CpG site a Spearman correlation with age, we filter out CpGs with inconsistent correlation signs between datasets and combine this information by taking the average. For blood we included the pseudo-bulked single-cell data to filter for opposite effect signs, but they are not contributing to the eventual site selection. Subsequently, we rank this list from highest to lowest absolute age correlating sites and prune this list for correlated sites. We do so by comparing the absolute correlation to age of sites within 5000 bases from each other, and if the age association is similar (absolute delta < 0.1), then we only keep the top age-associated site. The sites that are pruned away for the main age association are kept as backup sites and can be used when the main associated site is not available in a test sample. For the independent age associate sites, we then built up the methylation versus age matrix. To do so, we model DNAme per site given age, using a binomial model, and take the dataset of origin as a fixed effect covariate into the model, due to differences between datasets (Supplementary Fig. 12). We fit this model three times, once with linear age, once with log age and once with square root of age, to account for the potential non-linear relation between age and DNAme levels. We selected the best transformation per site based on the residual variation after fitting the GLM. Leveraging these models per CpG, we can calculate the DNAme values per age and, at the same time, correct these for observed dataset effect by adding back the average dataset effect. We chose to only model 1 week higher and lower as compared to observations in our combined training set.

### Predicting ages for new samples or cells

To model ages of new samples or cells, we select the overlapping sites between the clock sites and a new sample and compare the DNAme levels, similar to the procedure outlined in Trapp et al. With one minor difference, we directly calculate the absolute difference between the given DNAme value and the expected values and sum the log difference. This generalises the procedure proposed by Trapp et al. to also work on continuous DNAme profiles as observed in bulk data.

Lastly, we used cross-validation to select the number of age-associated DNAme sites to use when predicting epigenetic ages. During cross-validation, we used stratified folds based on dataset and cell type (if relevant) and dataset. We tested 50, 100, 250, 500, 750, 1000, 1250, 1500, 1750, and 2000 sites for training and found that 750 sites were optimal for both the blood and liver mode (Supplementary Fig. 8).

### Significance of the age deviation

To determine if an age prediction was significantly different from the expected chronological age we matched the real data to simulations matching the input cell and age. In detail, we select the predicted methylation profile matching the age of the cell and select the sites that are covered in the cell of interest. From this, we then simulate 1000 single cells, which, when combined, match the expected methylation profile derived from the expected matrix for the exact same sites covered in a cell of interest. Subsequently, we predict the ages of these simulated cells and can place the age prediction of the cell of interest in the distribution of random predictions. Based on this, we can calculate if the real cell is an outlier in terms of age prediction, defined as less than 5% of the permuted predictions are higher, or lower than the real prediction (empirical FDR 5%).

### Enrichment analyses

To test for gene set enrichment analyses we leveraged g:Profiler^62^, when assessing enrichments for DNAme we mapped promoters and enhancers to the closest gene and did the enrichment via g:Profiler. When leveraging g:Profiler we made sure the backgrounds are matched to the tested genes, instead of the default whole genome background.

To test for CpG enrichments in the epigenetic clocks, we leveraged a Fisher exact test. We selected all considered sites in the clocks (scEpiAge-blood, scEpiAge-liverclock, or the Stubbs clock) as a background and counted the number of times a site would be within any of these categories (CpG islands, CGI shores, CGI shelves, CGI inter, FANTOM5 enhancers, Repeats, Gene bodies, lncRNAs, CDS, Introns, Exons, Intergenic regions, 3’ UTRs, 5’ UTRs, TES, TSS, Promoters, CGI Promoters, Non-CGI Promoters), stratifying for a selected clock site or a background site. The MM10 annotation was derived from UCSC.

### Linking epigenetic age and number of mutations

To assess the link between epigenetic ageing, we used cellsnp-lite^63^ to call variants from the bam files. We used the default options to call variants either exclusive to the mitochondria or call variants genome wide. Next, we counted the number of sites that at least had one call of an alternative allele and used this as a proxy for the number of somatic mutations. To assess the link between the number of mutations and the epigenetic age, we leveraged a generalised linear model with a binomial link function, testing the link of the ratio of alternative containing sites and total number of sites called in a cell relative to epigenetic age. In the models, we included the scaled number of sequenced reads, genome-wide or MT-specific as covariates and added a random effect for cell type and mouse to account for mouse-to-mouse and cell type-to-cell type variability. The linear models were built in R using the LME4^61^ package.

### Including expressed genes into the ageing model

To try and link our observations on the RNA side and DNAme side of our work we produced one final ageing model. Here we choose to fit an elastic net regression model over the predicted scEpiAge of a cell combined with the number of expressed genes. For this, we used the glmnet R package (4.1)^64^ and conducted an 11-fold cross-validation, leaving one mouse out at a time, to account for relatedness between the cells from the same mouse.

### Statistics and reproducibility

No statistical method was used to predetermine sample size. The experiments were not randomised. The Investigators were not blinded to allocation during experiments and outcome assessment. No data were excluded from the analyses, except for datasets removed or trimmed during quality control as described in the respective sections above.

### Reporting summary

Further information on research design is available in the Nature Portfolio Reporting Summary linked to this article.

### Supplementary information

Supplementary Information

Peer Review File

Description of Additional Supplementary Files

Supplementary Data 1

Supplementary Data 2

Supplementary Data 3

Supplementary Data 4

Supplementary Data 5

Supplementary Data 6

Reporting Summary

### Source data

Source Data

## Data Availability

The scM&T-seq and bulk RRBS data generated in this study have been deposited in the NCBI Gene Expression Omnibus (GEO) database under accession code GSE225173. The corresponding raw data can be found in the NCBI Sequence Read Archive (SRA) under accession code PRJNA934287. Source data are provided with this paper.

## Code availability

Code (clock): https://github.com/EpigenomeClock/scEpiAge^65^. Code (study): https://github.com/EpigenomeClock/scAgingPaper.

## References

[1] Bocklandt, S. et al. Epigenetic predictor of age. *PLoS ONE* **6**, e14821 (2011). 21731603 10.1371/journal.pone.0014821 PMC3120753

[2] Horvath, S. DNA methylation age of human tissues and cell types. *Genome Biol.* **14**, R115 (2013). 24138928 10.1186/gb-2013-14-10-r115 PMC4015143

[3] Hannum, G. et al. Genome-wide methylation profiles reveal quantitative views of human aging rates. *Mol. Cell* **49**, 359–367 (2013). 23177740 10.1016/j.molcel.2012.10.016 PMC3780611

[4] Petkovich, D. A. et al. Using DNA methylation profiling to evaluate biological age and longevity interventions. *Cell Metab.* **25**, 954–960.e6 (2017). 28380383 10.1016/j.cmet.2017.03.016 PMC5578459

[5] Stubbs, T. M. et al. Multi-tissue DNA methylation age predictor in mouse. *Genome Biol.* **18**, 68 (2017). 28399939 10.1186/s13059-017-1203-5 PMC5389178

[6] Wang, T. et al. Epigenetic aging signatures in mice livers are slowed by dwarfism, calorie restriction and rapamycin treatment. *Genome Biol.* **18**, 57 (2017). 28351423 10.1186/s13059-017-1186-2 PMC5371228

[7] Lu, A. T. et al. Universal DNA methylation age across mammalian tissues. *Nat. Aging* **3**, 1144–1166 (2023). 37563227 10.1038/s43587-023-00462-6 PMC10501909

[8] Belsky, D. W. et al. Quantification of the pace of biological aging in humans through a blood test, the DunedinPoAm DNA methylation algorithm. *eLife* **9**, e54870 (2020). 32367804 10.7554/eLife.54870 PMC7282814

[9] Levine, M. E. et al. An epigenetic biomarker of aging for lifespan and healthspan. *Aging* **10**, 573–591 (2018). 29676998 10.18632/aging.101414 PMC5940111

[10] Lu, A. T. et al. DNA methylation GrimAge strongly predicts lifespan and healthspan. *Aging* **11**, 303–327 (2019). 30669119 10.18632/aging.101684 PMC6366976

[11] Jaffe, A. E. & Irizarry, R. A. Accounting for cellular heterogeneity is critical in epigenome-wide association studies. *Genome Biol.* **15**, R31 (2014). 24495553 10.1186/gb-2014-15-2-r31 PMC4053810

[12] Farlik, M. et al. Single-cell DNA methylome sequencing and bioinformatic inference of epigenomic cell-state dynamics. *Cell Rep.* **10**, 1386–1397 (2015). 25732828 10.1016/j.celrep.2015.02.001 PMC4542311

[13] Gravina, S., Dong, X., Yu, B. & Vijg, J. Single-cell genome-wide bisulfite sequencing uncovers extensive heterogeneity in the mouse liver methylome. *Genome Biol.* **17**, 150 (2016). 27380908 10.1186/s13059-016-1011-3 PMC4934005

[14] Luo, C. et al. Single-cell methylomes identify neuronal subtypes and regulatory elements in mammalian cortex. *Science* **357**, 600–604 (2017). 28798132 10.1126/science.aan3351 PMC5570439

[15] Smallwood, S. A. et al. Single-cell genome-wide bisulfite sequencing for assessing epigenetic heterogeneity. *Nat. Methods* **11**, 817–820 (2014). 25042786 10.1038/nmeth.3035 PMC4117646

[16] Hernando-Herraez, I. et al. Ageing affects DNA methylation drift and transcriptional cell-to-cell variability in mouse muscle stem cells. *Nat. Commun.* **10**, 4361 (2019). 31554804 10.1038/s41467-019-12293-4 PMC6761124

[17] Trapp, A., Kerepesi, C. & Gladyshev, V. N. Profiling epigenetic age in single cells. *Nat. Aging* **1**, 1189–1201 (2021). 36211119 10.1038/s43587-021-00134-3 PMC9536112

[18] Angermueller, C. et al. Parallel single-cell sequencing links transcriptional and epigenetic heterogeneity. *Nat. Methods* **13**, 229–232 (2016). 26752769 10.1038/nmeth.3728 PMC4770512

[19] Teo, Y. V., Webb, A. & Neretti, N. Single-cell transcriptomics of peripheral blood in the aging mouse. *Aging* **15**, 6–20 (2023). 10.18632/aging.204471 PMC9876630 36622281

[20] Phipson, B. et al. *propeller:* testing for differences in cell type proportions in single cell data. *Bioinformatics* **38**, 4720–4726 (2022). 36005887 10.1093/bioinformatics/btac582 PMC9563678

[21] Tabula Muris Consortium. A single-cell transcriptomic atlas characterizes ageing tissues in the mouse. *Nature* **583**, 590–595 (2020). 10.1038/s41586-020-2496-1 PMC8240505 32669714

[22] Yazar, S. et al. Single-cell eQTL mapping identifies cell type–specific genetic control of autoimmune disease. *Science* **376**, eabf3041 (2022). 35389779 10.1126/science.abf3041

[23] Frenk, S. & Houseley, J. Gene expression hallmarks of cellular ageing. *Biogerontology* **19**, 547–566 (2018). 29492790 10.1007/s10522-018-9750-z PMC6223719

[24] Meer, M. V., Podolskiy, D. I., Tyshkovskiy, A. & Gladyshev, V. N. A whole lifespan mouse multi-tissue DNA methylation clock. *eLife* **7**, e40675 (2018). 30427307 10.7554/eLife.40675 PMC6287945

[25] Reizel, Y. et al. Gender-specific postnatal demethylation and establishment of epigenetic memory. *Genes Dev.* **29**, 923–933 (2015). 25934504 10.1101/gad.259309.115 PMC4421981

[26] Thompson, M. J. et al. A multi-tissue full lifespan epigenetic clock for mice. *Aging* **10**, 2832–2854 (2018). 30348905 10.18632/aging.101590 PMC6224226

[27] Eirola, E., Doquire, G., Verleysen, M. & Lendasse, A. Distance estimation in numerical data sets with missing values. *Inf. Sci.* **240**, 115–128 (2013). 10.1016/j.ins.2013.03.043

[28] Han, Y. et al. Targeted methods for epigenetic age predictions in mice. *Sci. Rep.* **10**, 22439 (2020). 33384442 10.1038/s41598-020-79509-2 PMC7775437

[29] Han, Y. et al. New targeted approaches for epigenetic age predictions. *BMC Biol.* **18**, 71 (2020). 32580727 10.1186/s12915-020-00807-2 PMC7315536

[30] Karimzadeh, S. & Olafsson, S. Data clustering using proximity matrices with missing values. *Expert Syst. Appl.* **126**, 265–276 (2019). 10.1016/j.eswa.2019.02.022

[31] Cannon, M. V. et al. Maternal nutrition induces pervasive gene expression changes but no detectable DNA methylation differences in the liver of adult offspring. *PLoS ONE* **9**, e90335 (2014). 24594983 10.1371/journal.pone.0090335 PMC3940881

[32] Cole, J. J. et al. Diverse interventions that extend mouse lifespan suppress shared age-associated epigenetic changes at critical gene regulatory regions. *Genome Biol.* **18**, 58 (2017). 28351383 10.1186/s13059-017-1185-3 PMC5370462

[33] Alonso, C. et al. Metabolomic identification of subtypes of nonalcoholic steatohepatitis. *Gastroenterology* **152**, 1449–1461.e7 (2017). 28132890 10.1053/j.gastro.2017.01.015 PMC5406239

[34] Cagan, A. et al. Somatic mutation rates scale with lifespan across mammals. *Nature* **604**, 517–524 (2022). 35418684 10.1038/s41586-022-04618-z PMC9021023

[35] Martincorena, I. & Campbell, P. J. Somatic mutation in cancer and normal cells. *Science* **349**, 1483–1489 (2015). 26404825 10.1126/science.aab4082

[36] Moore, L. et al. The mutational landscape of human somatic and germline cells. *Nature* **597**, 381–386 (2021). 34433962 10.1038/s41586-021-03822-7

[37] López-Otín, C., Blasco, M. A., Partridge, L., Serrano, M. & Kroemer, G. Hallmarks of aging: an expanding universe. *Cell* **186**, 243–278 (2023). 10.1016/j.cell.2022.11.001 36599349

[38] López-Otín, C., Blasco, M. A., Partridge, L., Serrano, M. & Kroemer, G. The hallmarks of aging. *Cell* **153**, 1194–1217 (2013). 23746838 10.1016/j.cell.2013.05.039 PMC3836174

[39] Florea, M. Aging and immortality in unicellular species. *Mech. Ageing Dev.* **167**, 5–15 (2017). 28844968 10.1016/j.mad.2017.08.006

[40] Zou, Z. et al. A single-cell transcriptomic atlas of human skin aging. *Dev. Cell* **56**, 383–397.e8 (2021). 33238152 10.1016/j.devcel.2020.11.002

[41] Pappalardo, X. G. & Barra, V. Losing DNA methylation at repetitive elements and breaking bad. *Epigenetics Chromatin* **14**, 25 (2021). 34082816 10.1186/s13072-021-00400-z PMC8173753

[42] Ehrlich, M. DNA hypermethylation in disease: mechanisms and clinical relevance. *Epigenetics* **14**, 1141–1163 (2019). 31284823 10.1080/15592294.2019.1638701 PMC6791695

[43] Kabacik, S. et al. The relationship between epigenetic age and the hallmarks of aging in human cells. *Nat. Aging* **2**, 484–493 (2022). 37034474 10.1038/s43587-022-00220-0 PMC10077971

[44] Jung, H. et al. Thioredoxin-interacting protein regulates haematopoietic stem cell ageing and rejuvenation by inhibiting p38 kinase activity. *Nat. Commun.* **7**, 13674 (2016). 27929088 10.1038/ncomms13674 PMC5155146

[45] Macaulay, I. C. et al. Separation and parallel sequencing of the genomes and transcriptomes of single cells using G&T-seq. *Nat. Protoc.* **11**, 2081–2103 (2016). 27685099 10.1038/nprot.2016.138

[46] Clark, S. J. et al. Genome-wide base-resolution mapping of DNA methylation in single cells using single-cell bisulfite sequencing (scBS-seq). *Nat. Protoc.* **12**, 534–547 (2017). 28182018 10.1038/nprot.2016.187

[47] Linker, S. M. et al. Combined single-cell profiling of expression and DNA methylation reveals splicing regulation and heterogeneity. *Genome Biol.* **20**, 30 (2019). 30744673 10.1186/s13059-019-1644-0 PMC6371455

[48] Krueger, F. & Andrews, S. R. Bismark: a flexible aligner and methylation caller for Bisulfite-Seq applications. *Bioinformatics* **27**, 1571–1572 (2011). 21493656 10.1093/bioinformatics/btr167 PMC3102221

[49] Veeneman, B. A., Shukla, S., Dhanasekaran, S. M., Chinnaiyan, A. M. & Nesvizhskii, A. I. Two-pass alignment improves novel splice junction quantification. *Bioinformatics* **32**, 43–49 (2016). 10.1093/bioinformatics/btv642 PMC5006238 26519505

[50] Liao, Y., Smyth, G. K. & Shi, W. The R package Rsubread is easier, faster, cheaper and better for alignment and quantification of RNA sequencing reads. *Nucleic Acids Res.* **47**, e47 (2019). 30783653 10.1093/nar/gkz114 PMC6486549

[51] Liao, Y., Smyth, G. K. & Shi, W. featureCounts: an efficient general purpose program for assigning sequence reads to genomic features. *Bioinformatics* **30**, 923–930 (2014). 24227677 10.1093/bioinformatics/btt656

[52] Cunningham, F. et al. Ensembl 2022. *Nucleic Acids Res.* **50**, D988–D995 (2022). 34791404 10.1093/nar/gkab1049 PMC8728283

[53] McCarthy, D. J., Campbell, K. R., Lun, A. T. L. & Wills, Q. F. Scater: pre-processing, quality control, normalization and visualization of single-cell RNA-seq data in R. *Bioinformatics* **33**, 1179–1186 (2017). 10.1093/bioinformatics/btw777 PMC5408845 28088763

[54] de Graaf, C. A. et al. Haemopedia: an expression atlas of murine hematopoietic cells. *Stem Cell Rep.* **7**, 571–582 (2016). 10.1016/j.stemcr.2016.07.007 PMC5031953 27499199

[55] Zappia, L., Phipson, B. & Oshlack, A. Splatter: simulation of single-cell RNA sequencing data. *Genome Biol.* **18**, 174 (2017). 28899397 10.1186/s13059-017-1305-0 PMC5596896

[56] Aran, D. et al. Reference-based analysis of lung single-cell sequencing reveals a transitional profibrotic macrophage. *Nat. Immunol.* **20**, 163–172 (2019). 30643263 10.1038/s41590-018-0276-y PMC6340744

[57] Von Meyenn, F. Profiling DNA methylation in human naïve pluripotent stem cells. *Methods Mol. Biol*. **2416**, 157–180 (2022). 10.1007/978-1-0716-1908-7_11 34870836

[58] McInnes, L., Healy, J., Saul, N. & Großberger, L. UMAP: uniform manifold approximation and projection for dimension reduction. *Journal of open source software*. **3**, 861 (2018).

[59] Josse, J. & Husson, F. missMDA: a package for handling missing values in multivariate data analysis. *J. Stat. Softw*. **70**, 1–31 (2016).

[60] Lun, A. T. L., Bach, K. & Marioni, J. C. Pooling across cells to normalize single-cell RNA sequencing data with many zero counts. *Genome Biol.* **17**, 75 (2016). 27122128 10.1186/s13059-016-0947-7 PMC4848819

[61] Bates, D., Mächler, M., Bolker, B. & Walker, S. Fitting linear mixed-effects models using lme4. *J. Stat. Softw*. **67**, 1–48 (2015).

[62] Reimand, J., Kull, M., Peterson, H., Hansen, J. & Vilo, J. g:Profiler—a web-based toolset for functional profiling of gene lists from large-scale experiments. *Nucleic Acids Res.* **35**, W193–W200 (2007). 17478515 10.1093/nar/gkm226 PMC1933153

[63] Huang, X. & Huang, Y. Cellsnp-lite: an efficient tool for genotyping single cells. *Bioinformatics* **37**, 4569–4571 (2021). 33963851 10.1093/bioinformatics/btab358

[64] Friedman, J., Hastie, T. & Tibshirani, R. Regularization paths for generalized linear models via coordinate descent. *J. Stat. Softw.* **33**, 1–22 (2010). 20808728 10.18637/jss.v033.i01 PMC2929880

[65] Bonder, M. J. & Lammers, F. EpigenomeClock/scEpiAge: v1.0.1. Zenodo 10.5281/ZENODO.12521513 (2024).

