# Supplementary material for: scEpiAge: an age predictor highlighting single-cell ageing heterogeneity in mouse blood
Source: Nat Commun. 2024 Aug 31;15:7567. doi: 10.1038/s41467-024-51833-5 (PMC11366017; doi:10.1038/s41467-024-51833-5)
Supplement: Supplementary file 1 — Supplementary Information [file 41467_2024_51833_MOESM1_ESM.pdf]

Supplementary Material

scEpiAge - An Age Predictor Highlighting Single-cell Ageing Heterogeneity in Mouse Blood

Bonder et al.

Supplementary Data 2

Supplementary Figures 3

Supplementary Tables

Supplementary Data 1: Details of collected peripheral blood samples from mice spanning ages from 10 to 101 weeks.

Supplementary Data 2: Number of genes expressed changes with age in the Tabula Muris Senis (The Tabula Muris Consortium et al., 2020) and OneK1K (Yazar et al., 2022) datasets. Statistics presented are derived from a linear model, testing the relation between the number of expressed genes and aging. Shown are the estimate, and standard error P values, T statistics and Q values of the associations. In the model we corrected for batch, cell type (if relevant) and sex.

A: Tabula Muris senis SmartSeq2 replicating tissues.
B: Tabula Muris senis 10X replicating tissues.
C: OneK1K replication information.

Supplementary Data 3: Ageing associated genes. Summary statistics of the age associations as derived from MAST. Shown are the statistics of both the continuous, discrete and integrated models. MAST derives its association statistics from a generalised linear model, we corrected for batch, and number of expressed features.

A: Genes differentially expressed with chronological age in CD8+ T cells.
B: Genes differentially expressed with chronological age in B cells, including replication information in scEpiAge in B cells.
C: Genes differentially expressed between 101 w old and younger in CD4+ T cells.
D: Genes differentially expressed between 101 w old and younger in CD8+ T cells.
E: Genes differentially expressed between 101 w old and younger B cells.
F: Genes differentially expressed with scEpiAge in CD8+ T cells.

Supplementary Data 4: Ageing associated genes enrichments. g:Profiler results of the B cell associated ageing genes, specifically for the genes increasing with ageing. g:Profiler uses a Fisher's one-tailed test.

Supplementary Data 5: Age-related DNAme changes in both enhancers and promoters. To relate aggregated DNA-methylation levels to aging we used a linear model, correcting for mouse and sequencing depth, p-values shown are from a likelihood ratio test.

A: Enhancer results in CD4+ T cells.
B: Promoter results in CD4+ T cells.
C: Promoter results in B cells.

Supplementary Data 6: Details of all bulk data sets included

A: Sample information on the datasets used for the modelling epigenetic age for blood.
B: Sample information on the datasets used for the modelling epigenetic age for liver.

Supplementary Figure 1

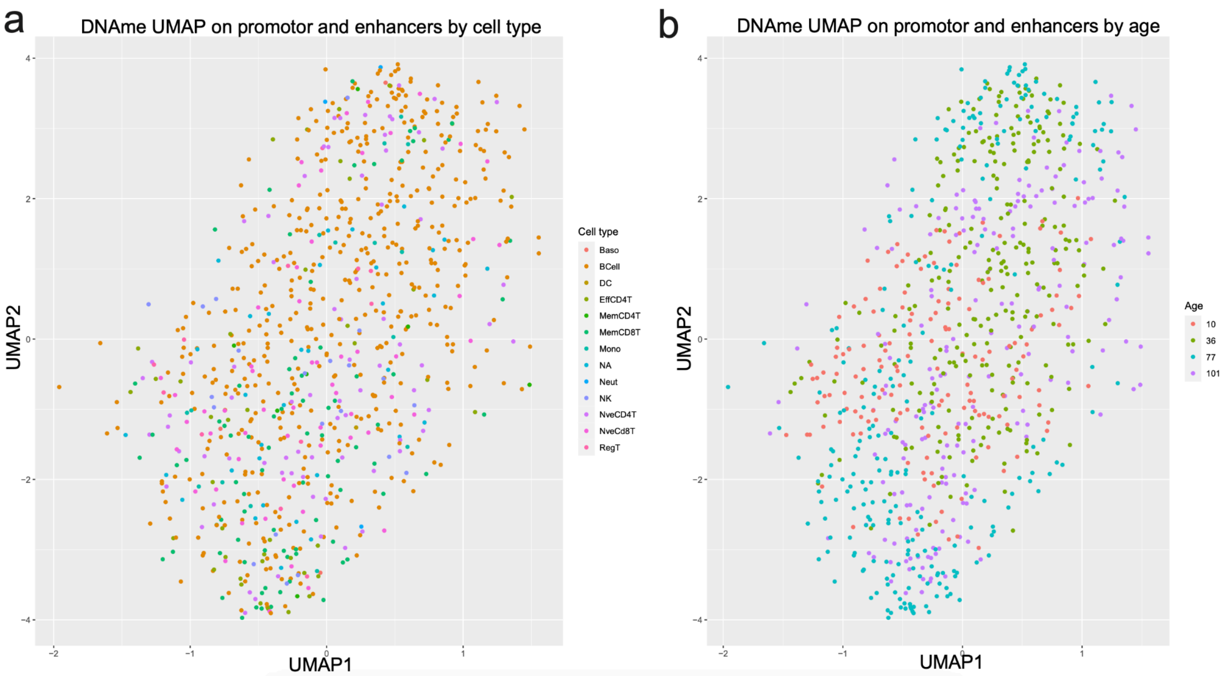

Supplementary Figure 1: DNAme did not show clear separations by age, cell type or animal. *Exploratory UMAP figures of the single cell DNAme data at enhancers and promoters: a) Cell type annotated UMAP (colours represent different cell types), and b) UMAP annotated by chronological age (colours represent different chronological ages).*

Supplementary Figure 2

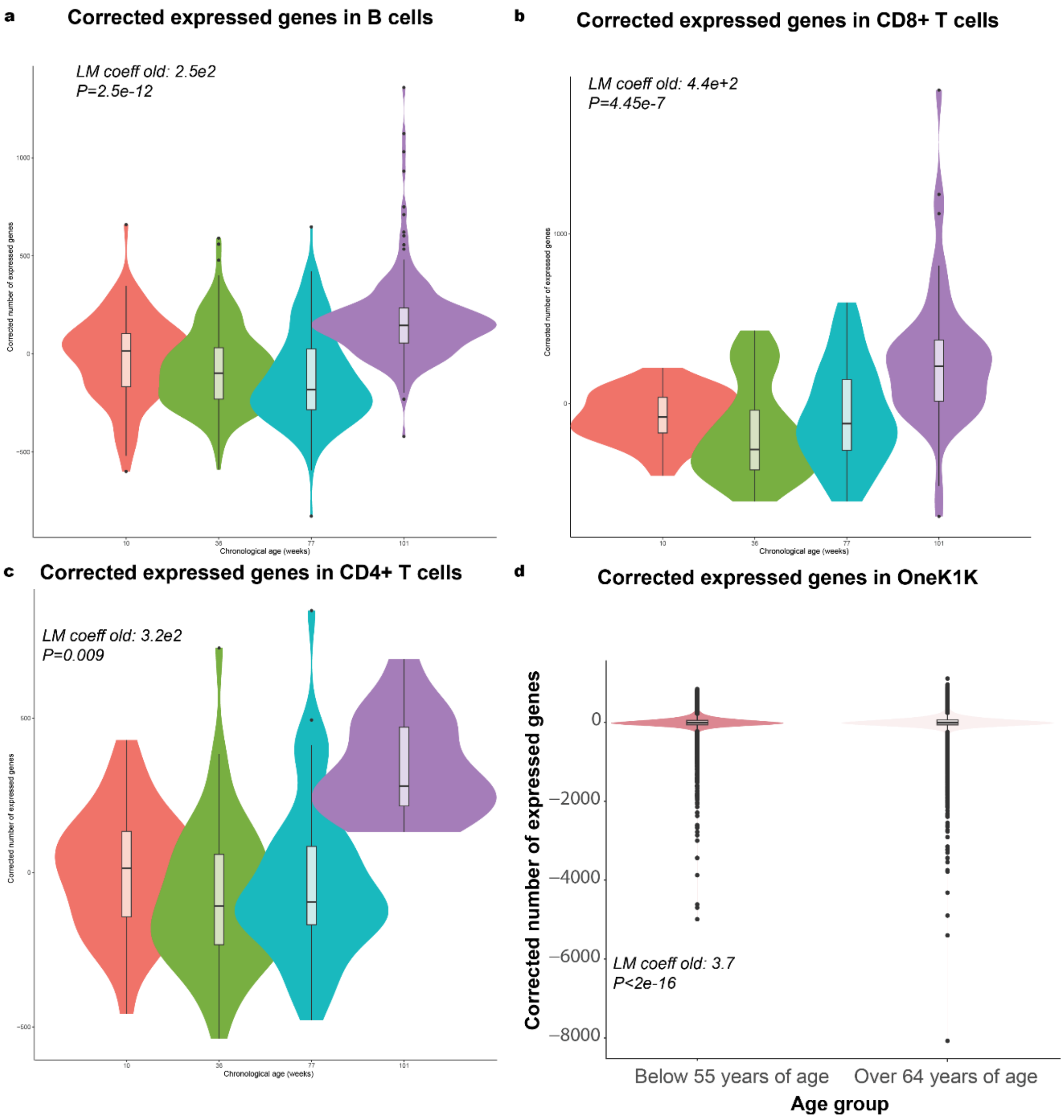

Supplementary Figure 2: Number of genes expressed per major cell type. *Violin plots showing the number of genes expressed/detected in each cell: a-c) Shown are the results for the major cell types analysed, namely a) B-cells, b) CD8+ T-cells, and c) CD4+ T-cells. d) Shown are the differences in a large human PBMC cohort, the OneK1K dataset (Yazar et al., 2022). To reflect the mouse analysis, we did split the humans data by age into samples below 55 years (roughly corresponds to below 77 weeks of age) and above 64 years (roughly corresponds to over 101 weeks of age in mice). Colours are only used to improve the visualisation. LM: linear model.*

Supplementary Figure 3

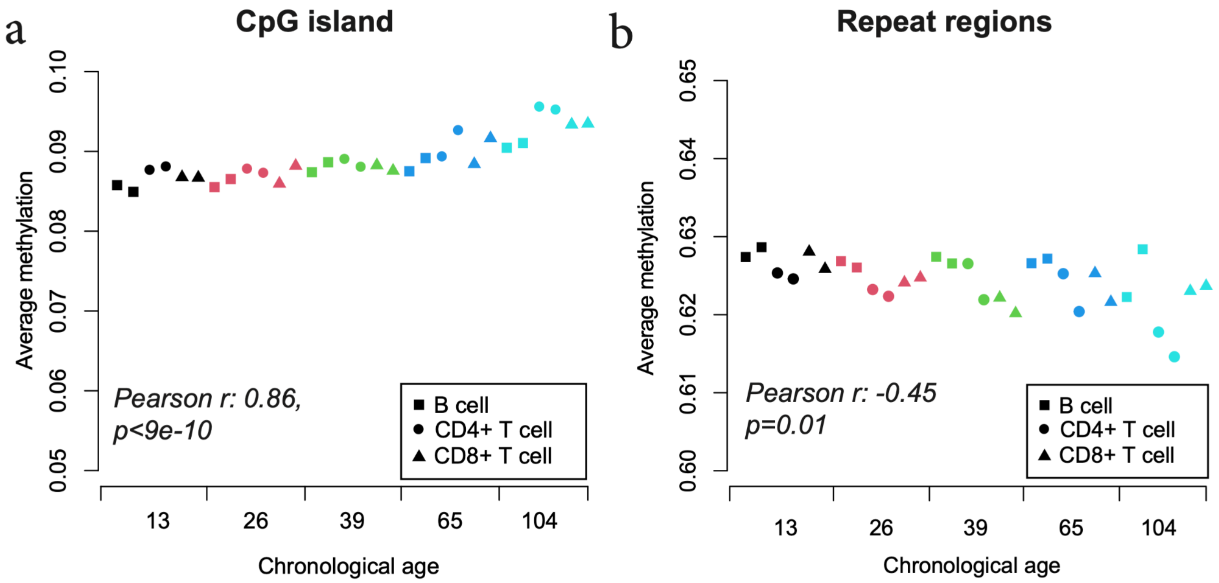

Supplementary Figure 3: scDNAme changes in the major cell types analysed. *Average single cell DNAme levels in a) CGIs and b) repeat regions. Cells are coloured and ordered by age. Shapes represent the cell types (squares B-cell, circles CD4+ T-cell, triangles CD8+ T-cell). Y-axis are scaled to the max and min values found in the presented data.*

Supplementary Figure 4

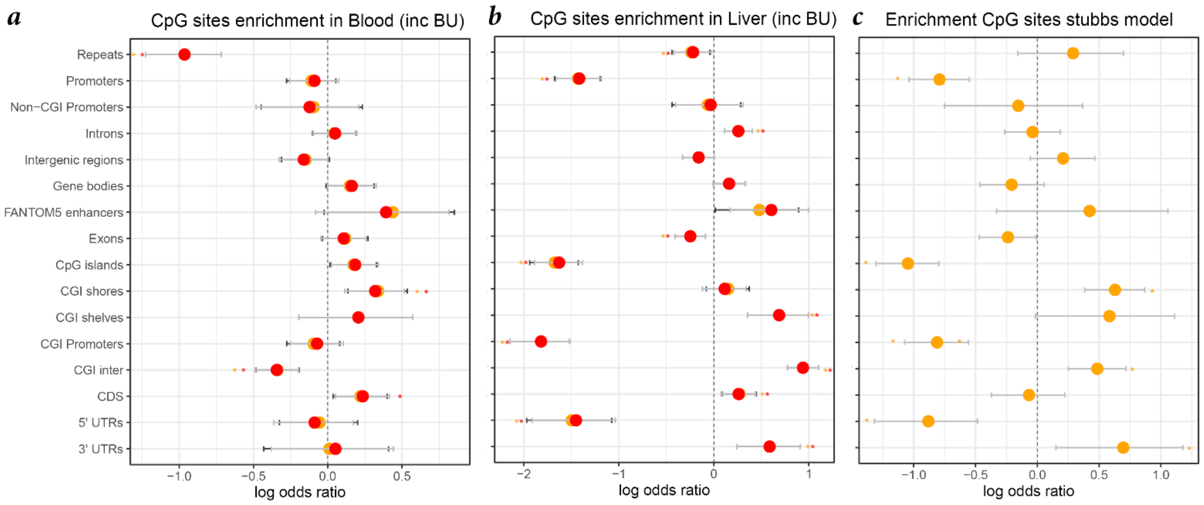

Supplementary* Figure 4: *Genomic enrichments of sites and back-up sites of the scEpiAge model. *We assessed the genomic enrichment of the sites (and back-up sites) selected in the a) scEpiAge blood and b) scEpiAge liver model, and compared them to the sites selected for the c) Stubbs et. al clock (Stubbs et al., 2017). CDS: coding sequence; UTR: untranslated region; CGI: CpG islands. In orange are the main sites, in red the backup sites.*

Supplementary Figure 5

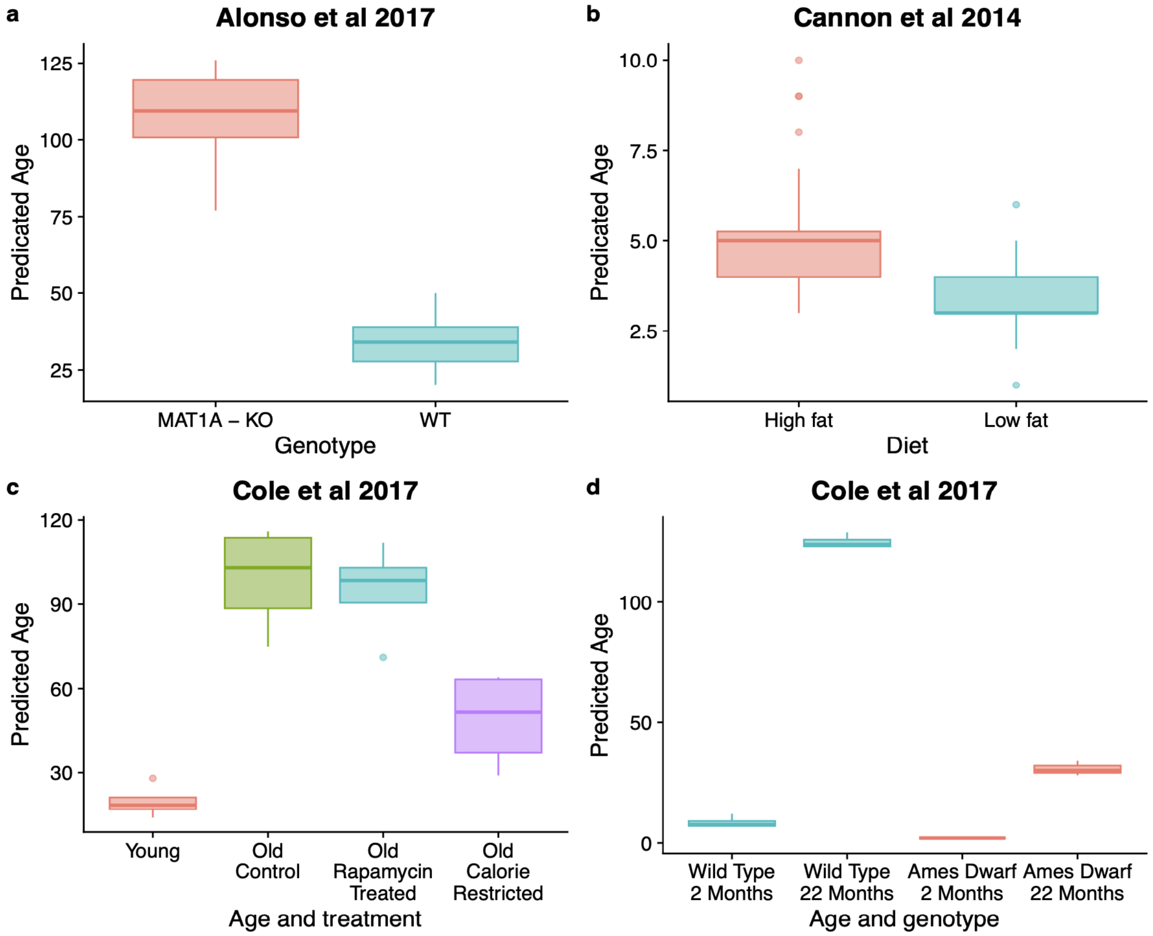

Supplementary Figure 5**: **scEpiAge measured in public DNAme datasets

a) Alonso et al. collected liver samples from 10 month old C57Bl/6 (WT control) and methionine adenosyltransferase 1a knockout (MAT1A-KO) mice, which spontaneously develop steatohepatitis and performed reduced representation bisulfite sequencing (RRBS-seq). We applied the scEpiAge model to these datasets and found an increase in predicted age in MAT1A-KO mice. b) Cannon et al. collected liver samples from mice fed a high fat or low fat diet and performed RRBS-seq. We applied the scEpiAge model to these datasets and found that a high fat diet increases the predicted epigenetic age compared to a low fat diet. c) Cole et al. collected liver samples from young (2 months), old untreated (22 months), old rapamycin treated (22s month, rapamycin treatment from 4 months of age) and old calorie restricted mice (22 months, CR initiated at 4 months of age) and performed whole-genome bisulfite sequencing (WGBS-seq). We applied the scEpiAge model to these datasets and could find beneficial effects of CR on the predicted age but not upon Rapamycin treatment. d) Cole et al. collected liver samples from young (2 months) and old (22 months) control and Ames Dwarf mice and performed WGBS-seq. We applied the scEpiAge model to these datasets and found that the Ames Dwarf mice epigenetically age at a slower pace as indicated by the scEpiAge predictions.

Colours are only used to improve the visualisation.

Supplementary Figure 6

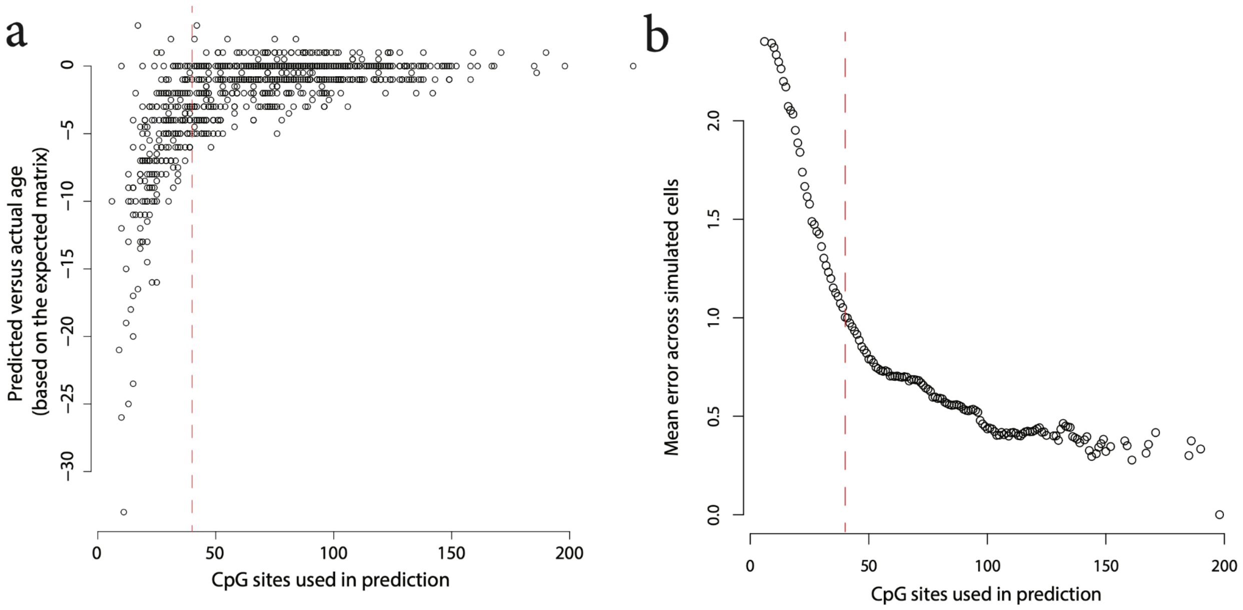

Supplementary Figure 6: Minimum overlapping sites needed for accurate scEpiAge predictions. *a) We calculated the difference between actual and predicted age in relation to the number of CpG sites used in prediction in simulated cells. For the expected data we matched the sites based on the actual cell. b) Additionally, we plotted the Average error of all simulated cells in relation to the number of CpG sites used in prediction.*

Supplementary Figure 7

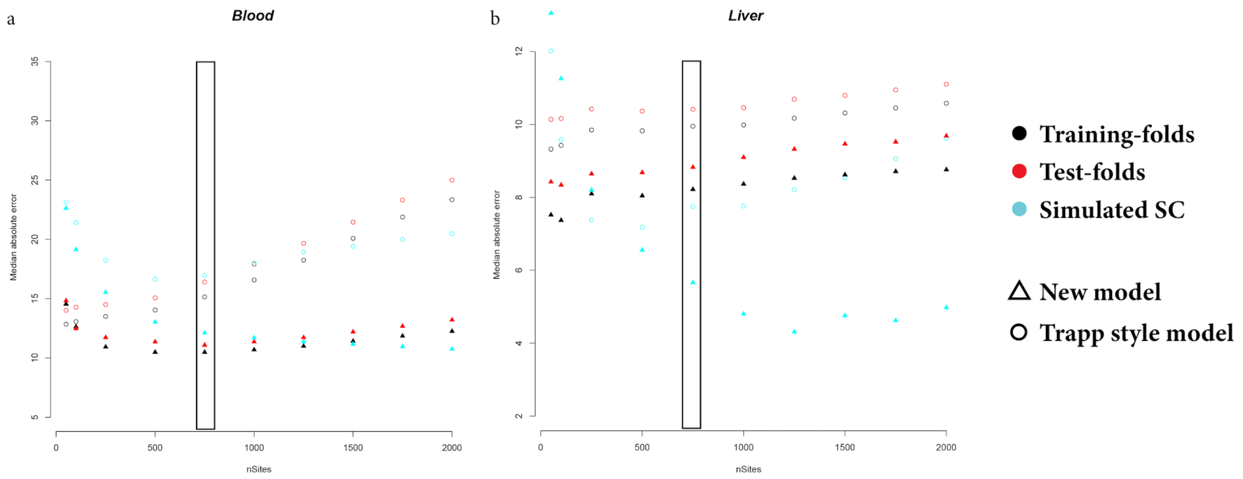

Supplementary Figure 7**: **Model performance during cross validation. a-b) *Error in cross validation when optimising the feature selection and number of sites. We show the performance on the training- and test-folds, as well as the performance on simulated single cells. In a) the performance of model building using the blood model, in b) the model setup in the Liver data setup. Due to improved feature selection and pruning of features the stability of the scEpiAge model increases relative to the original setup, and especially the performance on the simulated single cells is superior for the scEpiAge models. Shapes represent the model used (triangles scEpiAge; circles Trapp style model), colours represent the data type used (black training data, red test data, blue simulated single cells).*

Supplementary Figure 8

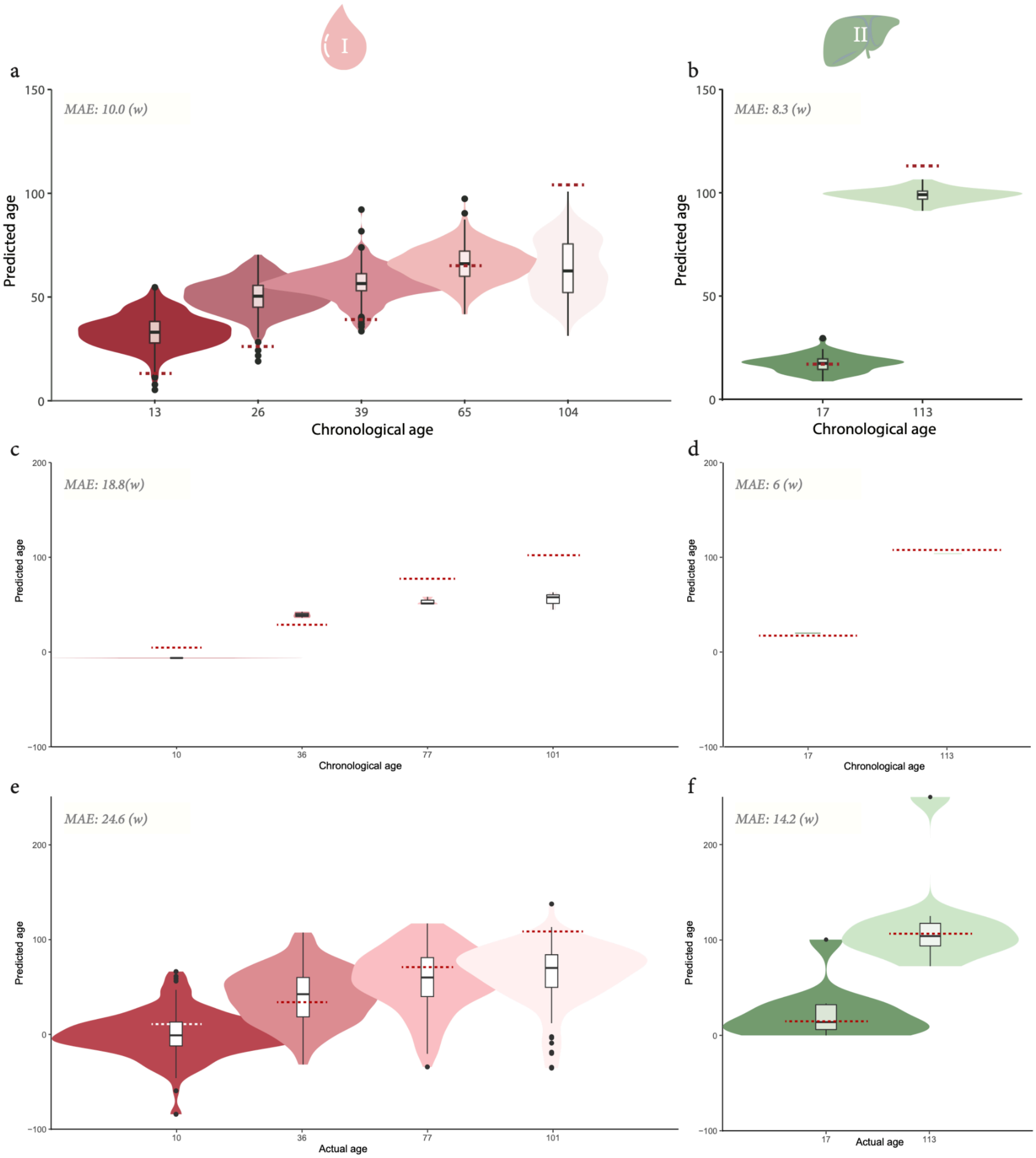

Supplementary Figure 8: Performance of the Trapp et. al model on scDNAme data. *We used the model described by Trapp et al. (Trapp et al., 2021) and assessed the performance of the model on simulated a) blood or b) liver single cells, pseudo bulked real c) blood and d) liver single cell data, and single cell e) blood and f) liver data. Colour shades are only used to improve the visualisation. MAE:* median absolute error. Created with BioRender.com released under a Creative Commons Attribution-NonCommercial-NoDerivs 4.0 International license.

Supplementary Figure 9

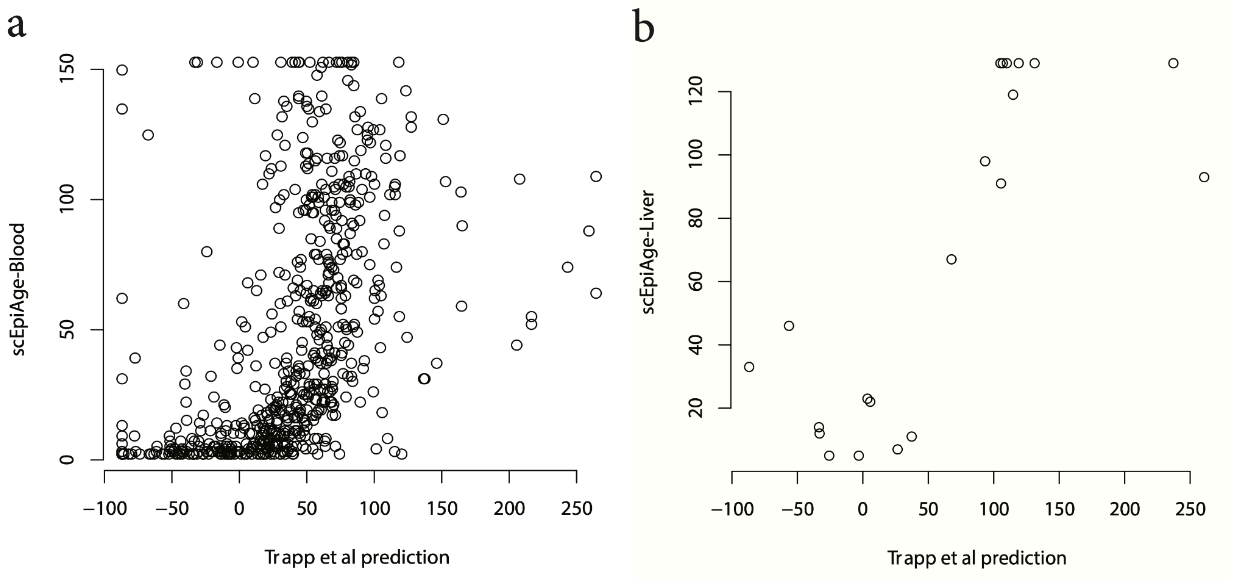

Supplementary Figure 9: Comparison between scEpiAge model and Trapp et al. model. *We used scEpiAge and the model from Trapp et al. (Trapp et al., 2021) to estimate the epigenetic age based on the DNAme data of all single cells. Shown are the correlation between both models for a) blood and b) liver single cell DNAme data.*

Supplementary Figure 10

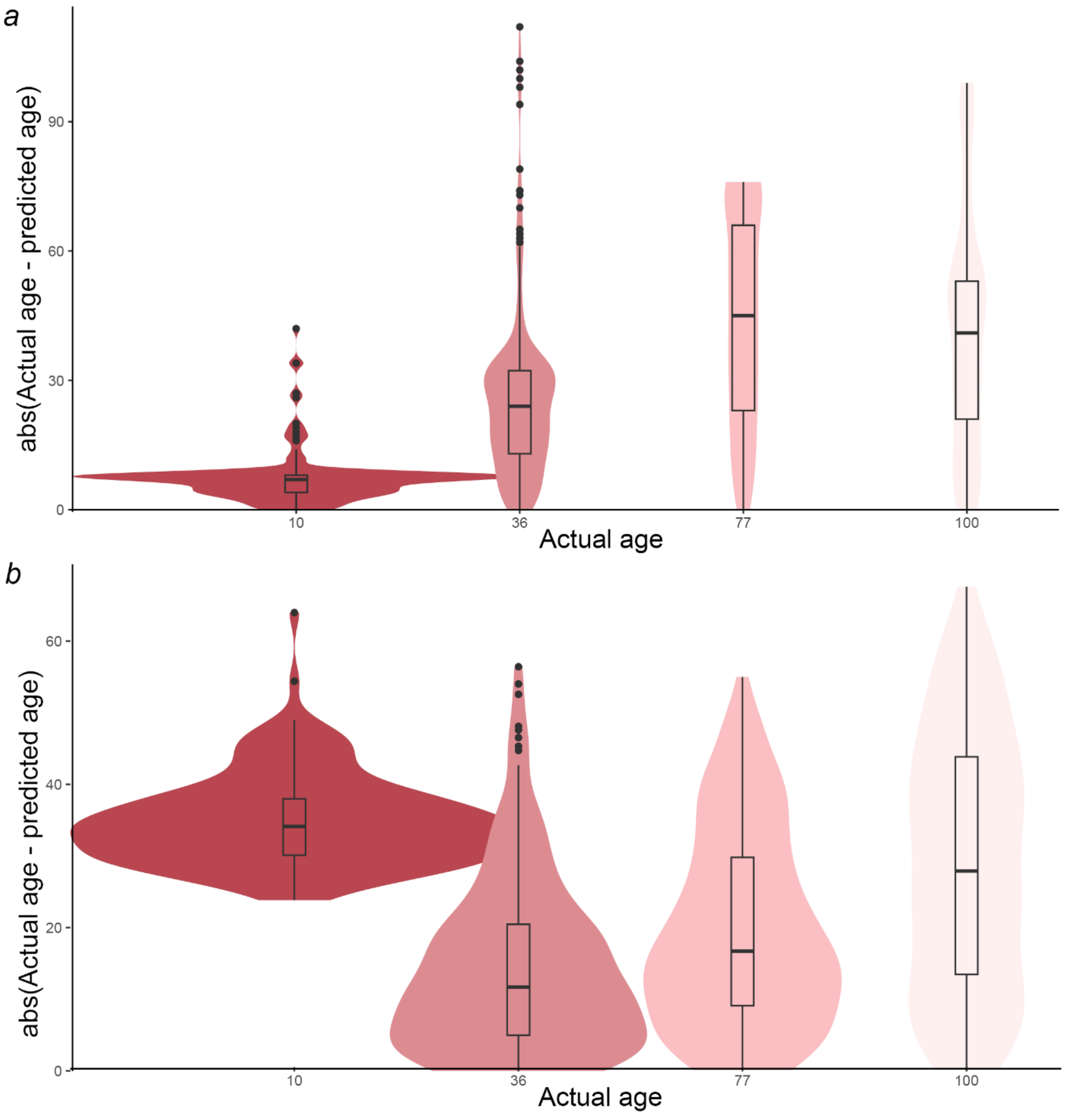

***Supplementary Figure 10: Delta ages of the single modal and multi model ageing models**. In a) the absolute delta age per age group is shown for the scEpiAge model, and in b) the absolute delta age is shown for the model built when also including the number of expressed genes. Interestingly the model including number of expressed genes has overall a better performance, median absolute deviation scEpiAge 26 weeks vs the multimodal model 21.5 weeks, but especially in the youngest mouse the median error is much lower in the DNAme lower model (median absolute deviation scEpiAge 7 weeks vs the multimodal model 34.1 weeks). Colour shades are only used to improve the visualisation.*

Supplementary Figure 11

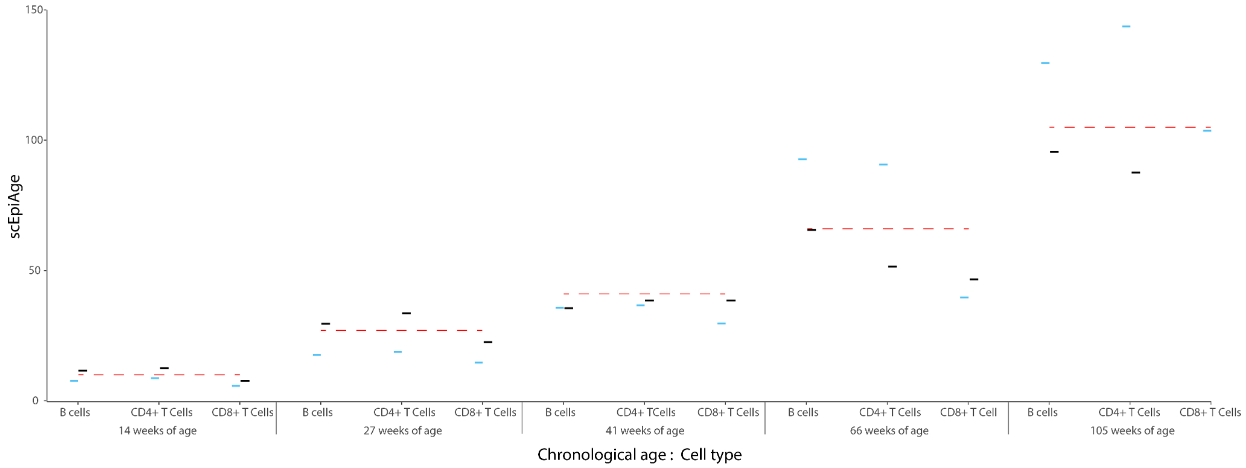

Supplementary Figure 11: Age prediction in sorted immune cells. *Shown are the age predictions in the sorted immune cell data in both the training and test data. Training data shown in black and left out test data in blue.*

Supplementary Figure 12

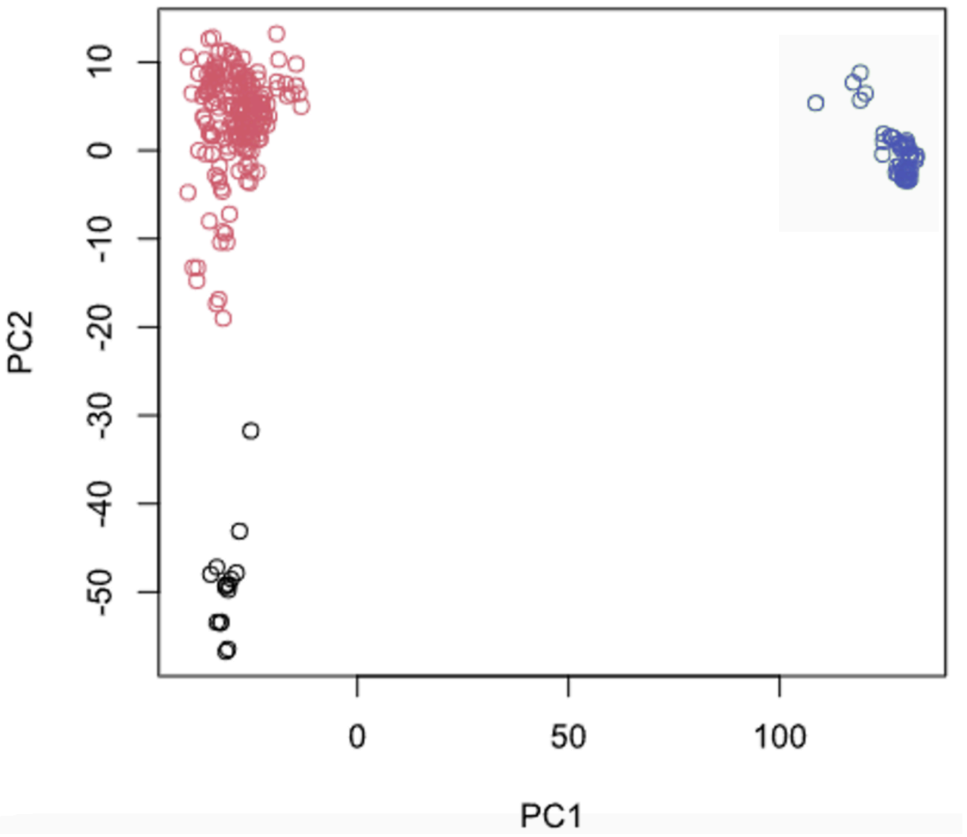

***Supplementary Figure 12**: **PCA on DNAme values over the three blood datasets used for the expected methylation matrix.** After dataset QC we do see substantial differences between the three main datasets used to construct the expected DNAme profiles, in red the Petkovic et al samples, in blue the Thompson et al samples and in black the new Babraham samples.*

Supplementary Figure 13

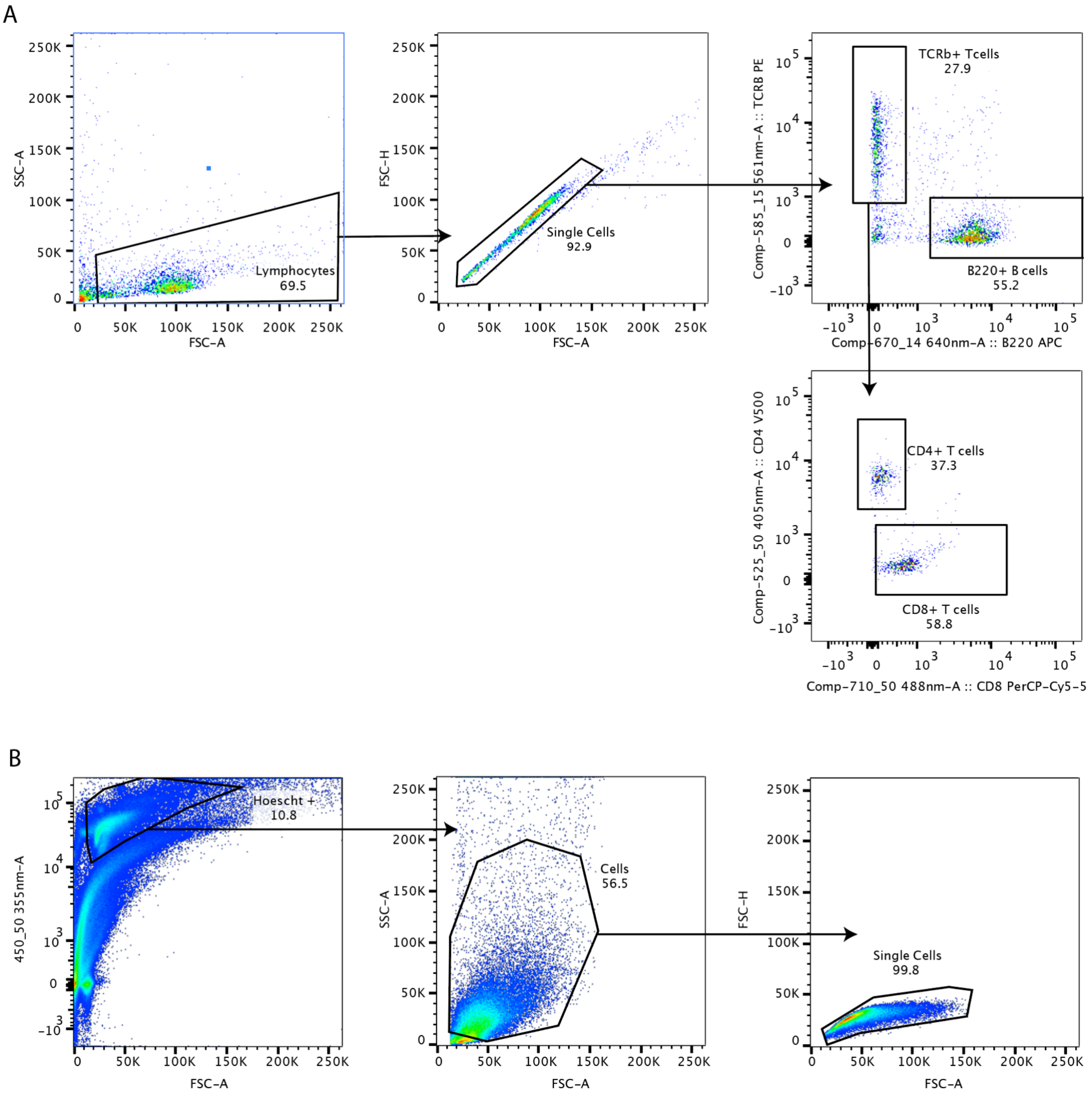

Supplementary Figure 13**: **FACS sequential gating/sorting strategy. *Shown are the gating strategies to enrich for a) B220+ B-Cells, CD4+ T-Cells, and CD8+ T-Cells, and b) collection of single nucleated cells from blood.*
