# Supplementary material for: scEpiAge: an age predictor highlighting single-cell ageing heterogeneity in mouse blood
Source: Nat Commun. 2024 Aug 31;15:7567. doi: 10.1038/s41467-024-51833-5 (PMC11366017; doi:10.1038/s41467-024-51833-5)
Supplement: Supplementary file 3 — Description of Additional Supplementary Files [file 41467_2024_51833_MOESM3_ESM.pdf]

Description of Additional Supplementary Files

Supplementary Data 1
Description: Details of collected peripheral blood samples from mice spanning ages from 10 to 101 weeks.

Supplementary Data 2
Description: Number of genes expressed changes with age in the Tabula Muris Senis (The Tabula Muris Consortium et al., 2020) and OneK1K (Yazar et al., 2022) datasets. Statistics presented are derived from a linear model, testing the relation between the number of expressed genes and aging. Shown are the estimate, and standard error P values, T statistics and Q values of the associations. In the model we corrected for batch, cell type (if relevant) and sex.

A: Tabula Muris senis SmartSeq2 replicating tissues.
B: Tabula Muris senis 10X replicating tissues.
C: OneK1K replication information.

Supplementary Data 3
Description: Ageing associated genes. Summary statistics of the age associations as derived from MAST. Shown are the statistics of both the continuous, discrete and integrated models. MAST derives its association statistics from a generalised linear model, we corrected for batch, and number of expressed features.

A: Genes differentially expressed with chronological age in CD8+ T cells.
B: Genes differentially expressed with chronological age in B cells, including replication information in scEpiAge in B cells.
C: Genes differentially expressed between 101 w old and younger in CD4+ T cells.
D: Genes differentially expressed between 101 w old and younger in CD8+ T cells.
E: Genes differentially expressed between 101 w old and younger B cells.
F: Genes differentially expressed with scEpiAge in CD8+ T cells.

Supplementary Data 4
Description: Ageing associated genes enrichments. g:Profiler results of the B cell associated ageing genes, specifically for the genes increasing with ageing. g:Profiler uses a Fisher's one-tailed test.

SSupplementary Data 5

Description: Age-related DNAme changes in both enhancers and promoters. To relate aggregated DNA-methylation levels to aging we used a linear model, correcting for mouse and sequencing depth, p-values shown are from a likelihood ratio test.

A: Enhancer results in CD4+ T cells.
B: Promoter results in CD4+ T cells.
C: Promoter results in B cells.

Supplementary Data 6

Description: Details of all bulk data sets included

A: Sample information on the datasets used for the modelling epigenetic age for blood.
B: Sample information on the datasets used for the modelling epigenetic age for liver.
