# Supplementary material for: scEpiAge: an age predictor highlighting single-cell ageing heterogeneity in mouse blood
Source: Nat Commun. 2024 Aug 31;15:7567. doi: 10.1038/s41467-024-51833-5 (PMC11366017; doi:10.1038/s41467-024-51833-5)
Supplement: Supplementary file 10 — Reporting Summary [file 41467_2024_51833_MOESM10_ESM.pdf]

nature portfolio

Corresponding author(s): Ferdinand von Meyenn & Marc Jan Bonder & Stephen J. Clark & Wolf Reik

Last updated by author(s): Aug 2, 2024

Reporting Summary

Nature Portfolio wishes to improve the reproducibility of the work that we publish. This form provides structure for consistency and transparency in reporting. For further information on Nature Portfolio policies, see our Editorial Policies and the Editorial Policy Checklist.

Statistics

For all statistical analyses, confirm that the following items are present in the figure legend, table legend, main text, or Methods section.

n/a	Confirmed	
☐	☒	The exact sample size (*n*) for each experimental group/condition, given as a discrete number and unit of measurement
☐	☒	A statement on whether measurements were taken from distinct samples or whether the same sample was measured repeatedly
☐	☒	The statistical test(s) used AND whether they are one- or two-sided<br>*Only common tests should be described solely by name; describe more complex techniques in the Methods section.*
☐	☒	A description of all covariates tested
☐	☒	A description of any assumptions or corrections, such as tests of normality and adjustment for multiple comparisons
☐	☒	A full description of the statistical parameters including central tendency (e.g. means) or other basic estimates (e.g. regression coefficient) AND variation (e.g. standard deviation) or associated estimates of uncertainty (e.g. confidence intervals)
☐	☒	For null hypothesis testing, the test statistic (e.g. *F*, *t*, *r*) with confidence intervals, effect sizes, degrees of freedom and *P* value noted<br>*Give P values as exact values whenever suitable.*
☒	☐	For Bayesian analysis, information on the choice of priors and Markov chain Monte Carlo settings
☒	☐	For hierarchical and complex designs, identification of the appropriate level for tests and full reporting of outcomes
☒	☐	Estimates of effect sizes (e.g. Cohen's *d*, Pearson's *r*), indicating how they were calculated

Our web collection on statistics for biologists contains articles on many of the points above.

Software and code

Policy information about availability of computer code

Data collection

Single cells - scM&T-seq
scM&T-seq data was processed as described previously (DNAme: (Angermueller et al., 2016); RNA: (Linker et al., 2019)). scBS-seq data was processed as described previously; briefly, reads had 6bp removed on their 5’-ends to remove the random primed portion of the reads, and were also adapter- and quality-trimmed using Trim Galore (v0.6.7; options --clip_r1 6). Trimmed reads were aligned to the bisulfite converted GRCm38 mouse genome using Bismark v0.22.3 in single-end mode (options: --non_directional) (Krueger and Andrews, 2011). Methylation calls were extracted after duplicate reads had been removed (deduplicate_bismark). scRNA-seq data was processed as described previously, briefly: the RNA-seq reads were adapter and quality-trimmed using Trim Galore (v0.6.7), and aligned to the GRCm38 mouse genome build using STAR (2.7.1a) (Veeneman et al., 2015), in two-pass mode. Expression quantification was performed leveraging FeatureCounts available in Subread 1.6 (Liao et al., 2019, 2014), and based on ENSEMBL version 96 (Cunningham et al., 2022).

Single cell quality control and normalisation
scM&T-seq data was quality controlled pr level. On the DNAme side We removed cells with a low read depth (removing cells with < 1M reads), removed cells with a low unique number of mapping reads (cells with < 50,000 uniquely aligned reads are removed), and high non-CpG methylation levels (cells with non-CpG meth >20% are removed). This left a total of 853 high quality cells. For downstream analysis we mapped all CpGs to the forward strand and removed non-CpG or ambiguous methylation calls.
Quality control of scRNA-seq data was performed using the SCATER package (McCarthy et al., 2017). Cells were retained for downstream analysis if they had at least 150,000 counts from endogenous genes, at least 1,500 genes with non-zero expression, less than 90% counts came from the top 100 highest expressed features and less than 15% mitochondrial reads. After quality control, 981 out of 1,055 blood single-cells were considered for downstream analysis. For further analyses, expression counts were SCRAN normalised into counts per million and log transformed (log(normCPM+1)).
During our analysis we found that a proportion of cells had very high expression values for haemoglobin genes (Hbb-bt, Hbb-bs, Hba-a1, and

Hba-a2), leveraging the expression counts of these genes we estimated the number of red blood (RB) cells contaminating our expression level. The expression of the four genes formed distinctive peaks, around no expression, around 5 reads per gene per cell and around 10 reads per gene per cell. We combined the information over the marker genes and defined cells with on average 5 reads per marker per cell as having 1 RB cell as contamination (348 cells), and if on average more than 10 reads per marker per cell was found we defined it as 2 contaminating RB cells (69).

Cell type annotation

We leveraged a combined de novo and reference-based mapping setup to annotate cells to cell types. Specifically, for the de novo we leveraged shared-nearest neighbour (SNN) from Seurat, as input we used the first 10 PCs, derived from the top 2,000 highly varying genes that are expressed in at least 1% of the cells, the SNN clustering resolution was set to 0.5. This yielded 12 clusters. In parallel we performed a reference-based cell annotation, to do so we took the bulk RNA-seq data from the haemopedia resource (de Graaf et al., 2016). The resource contains Bulk RNA-seq data of 57 different flow sorted mouse blood cell types, originating from 13 different cell lineages and are derived from healthy mice. The raw data was reprocessed with the same pipeline as our single cell data, see above. From this we simulated single cells leveraging Splatter (Zappia et al., 2017), expression counts were taken from the bulk data, other parameters needed for the simulation were derived from the actual sc-RNAseq data. By leveraging SingleR (Aran et al., 2019) we subsequently annotated the individual cells to the best matching simulated single cells from Heamopedia and annotated them to the relevant cell type. Lastly, we combine the information from SNN and SingleR and do a majority vote per cluster to assign final cell types per cluster and thereby cells. We leveraged the cell type annotation derived from RNA also in the DNAme analyses. Given the low cell numbers we focused the cell type specific analysis to B cells, CD4+ T cells (EffCD4T, MemCD4T, NveCD4T, RegT), and CD8+ T cells (MemCD8T, NveCd8T).

Bulk RRBS data processing and normalisation

RRBS and WGBS datasets were processed as described previously (Stubbs et al., 2017; von Meyenn, 2022), aligning reads to the bisulfite converted GRCm38 mouse genome using Bismark v0.22.3 (Krueger and Andrews, 2011). After mapping we transformed the DNAme calls to the forward direction and removed non-CpG or ambiguous methylation calls.

We performed per tissue (liver and blood) and dataset (Petkovich; Reizel; Meer; Thompson; Gravina; Babraham_p1, Babraham_p2, Babraham_p3) quality control. To do so we selected sites that had at least 5X coverage in 80% of the samples, we dropped samples with high missingness rate (>25%) and subsetted per dataset tissue combination to sites present in all samples. Based on this set we did a PCA, again per dataset tissue combination, and dropped outliers on PC1 and PC2, we defined outliers as samples with scores higher (or lower) than mean plus (or minus) two times SD on each of the two PCs.

Data analysis

Code (clock): https://github.com/EpigenomeClock/scEpiAge

Code (study): https://github.com/EpigenomeClock/scAgingPaper

UMAP on expression and DNAme

To get an overview of the global structures in both the gene expression data and the DNAme data we used UMAP (McInnes et al., 2018). On expression we used the top 5,000 most highly variable genes and use the “runUMAP” function from the SCATER (McCarthy et al., 2017) package with parameters “n_neighbors=8, min_dist=1” to make the UMAP plots (Fig 1c & 1d). On DNAme we joined the information on DNAme levels on promoters and enhancers, given the sparsity we removed regions with observations in less than 80% of the cells. Next we used the “imputePCA()” function from the missMDA (Josse and Husson, 2016) package to impute the missing information. Subsequently we selected the 25% most variable regions and made a UMAP on the 15 first PCs (Suppl Fig 1a & 1b).

Tissue cell composition analysis

To test for effects of age on cell type composition we used propeller (Phipson et al., 2022), implemented in the Specle R package. We transformed the proportions using the logit function, leveraging “getTranssformedProps()” and used the “propeller.annova()” function to test for the effect of age, and correct for multiple testing.

Differential expression analysis

We performed differential gene expression analysis leveraging MAST in the three major cell types (B-cell, CD4+ and CD8+ T-cells). Age, or epigenetic age, was used as a continuous variable and we treated, number of expressed genes, mouse ID, and Haemoglobin contamination as covariates. For T-cell analysis we also took sub-celltype proportion into account. For each of the three test cell types we filtered to: 1) protein coding genes, 2) genes expressed in at least 25% of the cells, selected the genes with a high biological variation (by measuring the “modelGeneVarByPoisson” function implemented in SCRAN (Lun et al., 2016) (var>1)), 3) and lastly only tested genes that are expressed in at least 5 cells in at least 2 distinct age groups. Additionally, we tested for a binary effect of ageing between the ages lower then 101 weeks versus the 101 week old cells.

The MAST model implements a hurdle model that combines information from two tests, a continuous model (for cells with non-zero expression levels), and a binary model (testing expressed vs not expressed). The effect sizes of the continuous and discrete part of the model are then combined to give one final output. We filtered these to not have significant results (nominal P<0.05 but in opposite direction between the tests) and performed Storey’s Qvalue to account for multiple testing. Significance was defined at 10% FDR.

Ageing affects numbers of genes expressed in mouse

To test the relation between age (and epigenetic age) and the number of genes expressed we used a linear model, implemented in R. For the test in our ageing mouse blood data we treated age as a binary variable (old: 101 weeks, young < 101 weeks) and corrected for number of expressed genes, cell types, and Haemoglobin contamination, unless otherwise specified.

For replication in the Tabula Muris data we mimicked the binary analysis testing ages lower or equal than 77 weeks versus ages higher than or equal to 101 weeks. Here again we corrected for the number of sequenced reads, and if appropriate corrected for sex. For the replication in the human PBMC data we matched these age criteria by selecting individuals below or equal to 55 years as young (roughly matching the 77 weeks in mouse) , and assigned individuals over 64 years of age as old (roughly matching the 101 weeks age in mouse). In oneK1K we corrected the number of genes expressed for the number of sequenced reads, material batch, and sequencing pool.

Differential methylation analysis

We performed the single cell differential DNAme analysis leveraging a generalised linear mixed effect model (GLMM) implemented in lme4 (Bates et al., 2015) and R. We leveraged a binomial link function to capture the binary nature of single cell DNAme data, and leveraged the random effect to capture the effects driven by mouse In this setup we tested for differentially methylated regions (DMRs) in enhancers and

promoters, in the three major cell classes (B, CD4+ T and CD8+ T cells). We tested regions with DNAme information in more than 25% of the cells per cell type. The enhancer and promoter information was derived from UCSC.

To associate DNAme levels at CpG-island and repeat regions to ageing we combined all CpG sites in the relevant region category, covered in at least one cell of each of the donor mice. To assess the effect of age on the overall methylation levels we used a spearman rank test. To replicate this finding in the bulk data we selected these same sites, per category, and tested for the same effect in the sorted blood data, again using a spearman rank test.

Modelling epigenetic age

To build an epigenetic clock that is able to deal with sparsity better as compared to standard elastic net regression models we leverage a direct distance-based model similar to the Trapp et al model and similar to genetic prediction models. We built these models based on the data of the Quality controlled RRBS samples as described above, the selection for CpGs is done independent from the QC procedure described above.

Building the expected age - methylation matrix

The first step we took was to build up an expected methylation versus age matrix. Todo so we combined published bulk RRBS blood (or liver) datasets, specifically new Babraham RRBS samples, GSE93957 (Stubbs et al., 2017), GSE80672 (Petkovich et al., 2017), GSE60012 (Reizel et al., 2015), GSE121141 (Meer et al., 2018), and GSE120137 (Thompson et al., 2018) and reprocessed the raw data as described above. Next we select CpG sites that are covered in at least 2 studies, have at most 25% missingness per covered dataset, and 33% maximum overall missingness in the combined study. In the blood dataset we included the pseudo bulked single cell data to inform the site selection on non-RRBS data. This leaves 366,250 CpG sites in blood and 753,296 CpG sites in liver.

After this initial selection we calculate per datasets and per CpG site a spearman correlation with age, we filter out CpGs with inconsistent correlation signs between datasets and combine this information by taking the average. For blood we again included the pseudobulked single cell data to filter for opposite effect signs, but they are not contributing to the eventual site selection. Subsequently we rank this list from highest to lowest absolute age correlating sites and prune this list for correlated sites. We do so by comparing the absolute correlation to age of sites within 5,000 bases from each other and if the age association is similar (absolute delta <0.1) then we only keep the top age associated site. The sites that are pruned away for the main age association but are kept as back-up sites, that can be used when the main associated site is not available in a test sample. For the independent age associate sites we then built up the methylation versus age matrix. To do so we model DNAme per site given age, using a binomial model, and take the dataset of origin as a 1-hot encoded fixed effect covariate along. We fit this model three times, once with linear age, once with log age and once with square root of age, to account for non-linearities that age has with DNAme values. We selected the best transformation per site based on the residual variation after fitting the GLM. Leveraging these models per CpG we can calculate the DNAme values per age, and correct these for observed dataset effect, here we chose to only model one week higher and lower as compared to observations in our combined training set.

Predicting ages for new samples or cells

To model ages of new samples or cells we select the overlapping sites between the clock sites and a new sample and compare the DNAme levels, similar to the procedure outlined in Trapp et al. With one minor difference, we directly calculate the absolute difference between the given DNAme value and the expected values, and sum the log difference. This generalises the procedure proposed by Trapp et al to also work on continuous DNAme profiles as observed in bulk data.

Lastly, we used cross validation to select the number of age associated DNAme to us when predicting epigenetic ages. During cross validation we used stratified folds based on dataset and cell type (if relevant) and dataset. We tested 50, 100, 250, 500, 750, 1000, 1250, 1500, 1750, 2000 sites for training, and found that 750 sites was optimal for both the blood and liver model.

Significance of the age deviation

To determine if an age prediction was significantly different from the expected chronological age we matched the real data to simulations matching the input cell and age. In detail, we select the predicted methylation profile matching the age of the cell, and select the sites that are covered in the cell of interest. From this we then simulate 1,000 single cells, which when combined match the expected methylation profile derived from the expected matrix for the exact same sites covered in a cell of interest. Subsequently we predict the ages of these simulated cells and can place the age prediction of the cell of interest in the distribution of random predictions. Based on this we can calculate if the real cell is an outlier in terms of age prediction, defined as less than 5% of the permuted predictions are higher, or lower than the real prediction (empirical FDR 5%).

Enrichment analyses

To test for gene set enrichment analyses we leveraged g:Profiler (Reimand et al., 2007), when assessing enrichments for DNAme we mapped promoters and enhancers to the closest gene and did the enrichment via g:Profiler. When leveraging g:Profiler we made sure the backgrounds are matched to the tested genes, instead of the default whole genome background.

To test for CpG enrichments in the epigenetic clocks we leveraged a fisher exact test. We selected all considered sites in the clocks (scEpiAge-blood, scEpiAge-liverclock, or the Stubbs clock) as a background and counted the number of times a site would be within any of these categories (CpG islands, CGI shores, CGI shelves, CGI inter, FANTOM5 enhancers, Repeats, Gene bodies, lncRNAs, CDS, Introns, Exons, Intergenic regions, 3' UTRs, 5' UTRs, TES, TSS, Promoters, CGI Promoters, Non-CGI Promoters), stratifying for a selected clock site or a background site. The MM10 annotation was derived from UCSC.

For manuscripts utilizing custom algorithms or software that are central to the research but not yet described in published literature, software must be made available to editors and reviewers. We strongly encourage code deposition in a community repository (e.g. GitHub). See the Nature Portfolio guidelines for submitting code & software for further information.

Data

Policy information about availability of data

All manuscripts must include a data availability statement. This statement should provide the following information, where applicable:
- Accession codes, unique identifiers, or web links for publicly available datasets
- A description of any restrictions on data availability
- For clinical datasets or third party data, please ensure that the statement adheres to our policy

Sequencing data: GSE225173 (https://www.ncbi.nlm.nih.gov/geo/query/acc.cgi?acc=GSE225173)

Research involving human participants, their data, or biological material

Policy information about studies with human participants or human data. See also policy information about sex, gender (identity/presentation), and sexual orientation and race, ethnicity and racism.

Reporting on sex and gender	N/A
Reporting on race, ethnicity, or other socially relevant groupings	N/A
Population characteristics	N/A
Recruitment	N/A
Ethics oversight	N/A

Note that full information on the approval of the study protocol must also be provided in the manuscript.

Field-specific reporting

Please select the one below that is the best fit for your research. If you are not sure, read the appropriate sections before making your selection.

[x] Life sciences [ ] Behavioural & social sciences [ ] Ecological, evolutionary & environmental sciences

For a reference copy of the document with all sections, see nature.com/documents/nr-reporting-summary-flat.pdf

Life sciences study design

All studies must disclose on these points even when the disclosure is negative.

Sample size	3 animals per age and approx 100 single cells per animal.
Data exclusions	No data exclusion was done - except for sequencing datasets that did not fulfill the QC as outlined in the manuscript.
Replication	All data was used for the analysis. For each time point / age we used 3 animals
Randomization	No randomization was done.
Blinding	No blinding was done.

Reporting for specific materials, systems and methods

We require information from authors about some types of materials, experimental systems and methods used in many studies. Here, indicate whether each material, system or method listed is relevant to your study. If you are not sure if a list item applies to your research, read the appropriate section before selecting a response.

Materials & experimental systems

n/a	Involved in the study
☐	☒ Antibodies
☒	☐ Eukaryotic cell lines
☒	☐ Palaeontology and archaeology
☐	☒ Animals and other organisms
☒	☐ Clinical data
☒	☐ Dual use research of concern
☒	☐ Plants

Methods

n/a	Involved in the study
☒	☐ ChIP-seq
☐	☒ Flow cytometry
☒	☐ MRI-based neuroimaging

Antibodies

Antibodies used	anti-mouse TCR β chain (clone H57-597; Biolegend #109207), anti-mouse CD4 (clone GK1.5; Biolegend #100449), anti-mouse CD8a (clone 53-6.7; Biolegend #100759) and anti-mouse B220 (clone RA3-6B2; Biolegend #103211). All antibodies were used at a dilution of 1:300.
Validation	These are routinely used Antibodies by many immunological research groups and have been validated beforehand in the lab and by colleagues.

Animals and other research organisms

Policy information about studies involving animals; ARRIVE guidelines recommended for reporting animal research, and Sex and Gender in Research

Laboratory animals	Mice were bred and maintained in the Babraham Institute Biological Services Unit (BI BSU) under Specific Opportunistic Pathogen Free (SOPF) conditions. C57BL/6 J mice (supplied by Charles River Laboratories) were imported into the BI BSU by embryo transfer and bred there (/Babr) as a SOPF colony in plastic film isolators.
Wild animals	N/A
Reporting on sex	All animals were male.
Field-collected samples	N/A
Ethics oversight	This research complies with all relevant ethical regulations. Animal experiments were performed according to the UK Animals (Scientific Procedures) Act 1986 and approved by the Babraham Research Campus Animal Welfare and Ethical Review Body.

Note that full information on the approval of the study protocol must also be provided in the manuscript.

Plants

Seed stocks	N/A
Novel plant genotypes	N/A
Authentication	N/A

Flow Cytometry

Plots

Confirm that:

☒ The axis labels state the marker and fluorochrome used (e.g. CD4-FITC).

☒ The axis scales are clearly visible. Include numbers along axes only for bottom left plot of group (a 'group' is an analysis of identical markers).

☒ All plots are contour plots with outliers or pseudocolor plots.

☒ A numerical value for number of cells or percentage (with statistics) is provided.

Methodology

Sample preparation	Sorted immune cell types (CD4+, CD8+ T-cells, and B220+ B-cells) were collected from the same animals used in the Stubbs et al study 5. Briefly, samples were incubated with anti-mouse TCR β chain (clone H57-597; Biolegend #109207), anti-mouse CD4 (clone GK1.5; Biolegend #100449), anti-mouse CD8a (clone 53-6.7; Biolegend #100759) and anti-mouse B220 (clone RA3-6B2; Biolegend #103211). All antibodies were used at a dilution of 1:300. Cells were then washed once in PBS with 0.5% BSA, and flow sorted (BD FACSAria III cell sorter) directly into RLT plus lysis buffer (Qiagen #1053393).<br>Blood samples for single cell analysis were collected and directly processed. Red blood cells were lysed using 2 rounds of red blood cell lysis (RBC Lysis Buffer, Roche #11814389001), followed by 3 rounds of washes with PBS with 0.5% BSA. Cells were then stained with Hoechst 33342 (Thermo Fisher Scientific #H3570) and flow sorted (Supplementary Fig. 13b) into 96 well plates containing 2.5ul of RLT plus lysis buffer (Qiagen #1053393).
Instrument	BD FACSAria III cell sorter
Software	BD FACSAria III cell sorter software
Cell population abundance	Indicated in the plots
Gating strategy	Lymphocytes were gated based on forward and side scatter, then gated on singlets. T-cells were selected by gating for TCRβ+ and then gating for CD4+ or CD8a+. B-cells were selected by gating for TCRβ- cells and then gated for B220+ cells.<br>Single cells were gated on Hoechst+, FSC-A vs SSC-A and then FSC-A vs FSC-H to ensure nucleated single cells were deposited in each well.

☒ Tick this box to confirm that a figure exemplifying the gating strategy is provided in the Supplementary Information.
