# Supplementary material for: scEpiAge: an age predictor highlighting single-cell ageing heterogeneity in mouse blood
Source: Nat Commun. 2024 Aug 31;15:7567. doi: 10.1038/s41467-024-51833-5 (PMC11366017; doi:10.1038/s41467-024-51833-5)
Supplement: Supplementary file 2 — Peer Review File [file 41467_2024_51833_MOESM2_ESM.pdf]

This PDF Package contains multiple files grouped together within a single container PDF file.

Kofax Power PDF can be used to view, create and manage the files within this container.

To get started, click any file in the PDF Portfolio to view it.

- PDF Packages group multiple files and file types together to make them easier to distribute or share.
- PDF Packages allow you to organize related files for easier viewing and printing
- PDF Packages maintain the original security settings of the files or folders included in the container

Note: In older viewers/editors the files are stored as attachments to this page.

KOFAX
